# FgSnt1 of the Set3 HDAC complex plays a key role in mediating the regulation of histone acetylation by the cAMP-PKA pathway in *Fusarium graminearum*

**DOI:** 10.1371/journal.pgen.1010510

**Published:** 2022-12-07

**Authors:** Chen Gong, Daiying Xu, Daiyuan Sun, Jiangang Kang, Wei Wang, Jin-Rong Xu, Xue Zhang

**Affiliations:** 1 State Key Laboratory of Crop Stress Biology for Arid Areas, College of Plant Protection, Northwest A&F University, Yangling, China; 2 Department of Botany and Plant Pathology, Purdue University, West Lafayette, Indiana, United States of America; Oregon State University, UNITED STATES

## Abstract

The cAMP-PKA pathway is critical for regulating growth, differentiation, and pathogenesis in fungal pathogens. In *Fusarium graminearum*, mutants deleted of *PKR* regulatory-subunit of PKA had severe defects but often produced spontaneous suppressors. In this study eleven *pkr* suppressors were found to have mutations in *FgSNT1*, a component of the Set3C histone deacetylase (HDAC) complex, that result in the truncation of its C-terminal region. Targeted deletion of the C-terminal 98 aa (CT98) in *FgSNT1* suppressed the defects of *pkr* in growth and H4 acetylation. CT98 truncation also increased the interaction of FgSnt1 with Hdf1, a major HDAC in the Set3 complex. The *pkr* mutant had no detectable expression of the Cpk1 catalytic subunit and PKA activities, which was not suppressed by mutations in *FgSNT1*. Cpk1 directly interacted with the N-terminal region of FgSnt1 and phosphorylated it at S443, a conserved PKA-phosphorylation site. CT98 of FgSnt1 carrying the S443D mutation interacted with its own N-terminal region. Expression of *FgSNT1*^S443D^ rescued the defects of *pkr* in growth and H4 acetylation. Therefore, phosphorylation at S443 and suppressor mutations may relieve self-inhibitory binding of FgSnt1 and increase its interaction with Hdf1 and H4 acetylation, indicating a key role of FgSnt1 in crosstalk between cAMP signaling and Set3 complex.

## Author summary

The cAMP-protein kinase A (PKA) pathway is one of the well-conserved signal transduction pathways in *F*. *graminearum* that regulates various infection and developmental processes. In this study we showed that deletion of the C-terminal region of FgSnt1 suppressed the defects of the *pkr* mutants deleted of the regulator subunit of PKA in growth and H4 histone acetylation. Snt1 is a component of the well-conserved Set3 histone deacetylase (HDAC) complex that is important for regulating histone acetylation and gene expression. Truncation of FgSnt1 increased its interaction with Hdf1, a major HDAC in the Set3 complex. Although Pkr did not, the Cpk1 catalytic subunit of PKA directly interacted with the N-terminal region of FgSnt1 and phosphorylated it at the conserved S443 site. Nevertheless, Pkr is important for protecting the Cpk1 proteins from degradation by the 26S proteosome. The S443D mutation mimicking phosphorylation of FgSnt1 by Cpk1 enabled the likely self-inhibitory interaction between its N-terminal and C-terminal regions and partially rescued the defects of *pkr* and H4 acetylation. Overall, this work described the comprehensive genetic interaction of the Set3 complex with the cAMP-PKA pathway. Our work extends and deepens our understanding of cAMP-PKA pathway in histone acetylation and virulence of plant pathogenic fungi.

## Introduction

The filamentous ascomycete *Fusarium graminearum* is the major causal agent of Fusarium head blight (FHB), which is one of the most important diseases of wheat and barley worldwide [1]. It overwinters on plant debris and releases ascospores from perithecia in the spring. As the primary inoculum, ascospores initiate the disease cycle after landing on flowering wheat or barley heads and form infection cushions or compound appressoria for plant penetration. Morphologically distinct infectious hyphae can grow inter- and intra-cellularly in infected plant tissues and spread via the rachis from the initial infection site to neighboring spikelets [2,3]. Under favorable environmental conditions, outbreaks of FHB cause severe yield losses and often contaminate infested grains with the harmful mycotoxins deoxynivalenol (DON) and zearalenone [4]. DON is also an important virulence factor that facilitates the spread of *F*. *graminearum* infection from the initial infection site via the rachis to the rest of spikelets [4,5].

Unlike many other plant pathogenic ascomycetes, the genome of *F*. *graminearum* has no active transposable elements and contains less than 0.05% repetitive sequences. In addition, it has chromosomal regions that have higher genetic variations and are enriched with fungal-plant interaction-related genes [6]. Further studies showed that these chromosomal regions are correlated to facultative heterochromatin regions with high levels of H3K27me3 in vegetative hyphae [7,8]. Therefore, chromosomal organization and chromatin modification appear to play a critical role in regulating genes that are specifically expressed during different developmental and infection stages in *F*. *graminearum*. Consistent with the chromosomal organizations, the *TBL1* gene encoding a transducing-like protein homologous to yeast Sif2 was found to be important for conidiation and infectious growth in the rachis tissues [9]. Sif2 is a component of the well-conserved Set3 histone deacetylase (HDAC) complex that also consists of Set3, Snt1, Hos2, Cpr1, Hst1, and Hos4 in yeast [10]. In the rice blast fungus *Magnaporthe oryzae*, genes orthologous to the key components of the yeast Set3 complex are also important for conidiation, appressorium penetration, and invasive growth [11]. In *F*. *graminearum*, *HDF1*, an ortholog of yeast *HOS2*, is the major class II HDAC gene that plays a critical role in regulating hyphal growth, sexual and asexual reproduction, DON biosynthesis, and plant infection [12]. Recently, another HDAC gene, *FgRPD3*, and the *FgESA1* histone deacetylase (HAT) gene were found to play opposing roles in balancing histone acetylation and regulating transcriptional accessibility, growth, development, and infection processes in this fungal pathogen [13].

*F*. *graminearum* is a homothallic fungus that is amenable to classic and molecular genetic studies. Since the publication of its genome sequence, many genes with various biological functions, including those encoding protein kinases, transcription factors, and key components of intracellular signaling pathways, have been characterized for their functions in regulating infection, sexual and asexual reproduction, and DON production [14–19]. Although accelerated by the release of the *F*. *graminearum* genome, a substantial amount of functional work had already been accomplished. Like in other fungal pathogens [20,21], the cAMP-protein kinase A (PKA) pathway is one of the well-conserved signal transduction pathways in *F*. *graminearum* that regulates various infection processes [22,23]. The Fac1 adenylate cyclase and Pde1 cAMP phosphodiesterase responsible for regulating the intracellular cAMP level are the first two components of this important signaling pathway characterized for their functions in vegetative growth, conidiation, ascosporogenesis, and DON production in this pathogen [24,25]. The *CPK1* and *CPK2* genes encode the major and minor catalytic subunits of PKA, respectively, as well as the *PKR* gene encoding the regulatory subunit also have been functionally characterized in *F*. *graminearum* [22,23]. Whereas deletion of *CPK1* results in pleiotropic defects, the *cpk2* mutant has no detectable phenotypes. However, the *cpk1 cpk2* double mutant has more severe defects than the *cpk1* mutant [23]. The *pkr* mutant shares similar phenotypes with the *cpk1 cpk2* double mutant, including severe defects in growth and pathogenesis as well as loss of fertility, but the latter produced normal conidia. In contrast, conidia of *pkr* mutant have morphological defects and autophagy-related cell death [22].

Interestingly, both the *cpk1 cpk2* double and *pkr* deletion mutants of *F*. *graminearum* were unstable and produced spontaneous suppressor strains that had faster growth rate. All but one of the 30 spontaneous suppressor strains of the *cpk1 cpk2* mutant had null mutations in *FgSFL1* [26,27]. In the budding yeast *Saccharomyces cerevisiae*, Sfl1 is a transcription factor that functions downstream from cAMP signaling and interacts with the Cyc8-Tup1 co-repressor complex for regulating gene expression [28,29]. In the rice blast fungus *M*. *oryzae*, suppressor mutations in *MoSFL1* rescue the growth defect of the *cpkA cpk2* mutant by relieving the suppression of subsets of genes by the MoCyc8-Tup1 complex that normally requires the phosphorylation of MoSfl1 by PKA [26]. For the *pkr* mutant in *F*. *graminearum*, a total of 67 suppressor strains were isolated. Like suppressors of the *cpk1 cpk2* mutant, suppressors of the *pkr* mutant were rescued in growth defects but still blocked in sexual reproduction and spreading infectious growth via the rachis [22,23]. Whereas none of the suppressor strains of the *cpk1 cpk2* mutant had mutations in *PKR*, 12 of the *pkr* suppressors had mutations in *CPK1* that affects its PKA activities [22]. However, suppressor mutations in the remaining 55 suppressor strains of the *pkr* mutant remain to be identified.

To further characterize the cAMP-PKA pathway and suppressors of *pkr* in *F*. *graminearum*, in this study we identified mutations in *FgSNT1*, a core component of the Set3C HDAC complex, in 11 suppressor strains of the *pkr* mutant. All of these suppressor mutations resulted in the truncation of the C-terminal 275 or 98 (CT98) amino acids of *FgSNT1*. CT98 of FgSnt1 had self-inhibitory binding with its N-terminal region, which was relieved by the S443D or suppressor mutations. Targeted deletion of CT98 suppressed the defects of *pkr* in growth and H4 acetylation and increased the interaction of FgSnt1 with Hdf1, a HDAC of the Set3 complex. Taken together, these results indicated that FgSnt1 plays a key role in the functional relationship between the cAMP-PKA pathway and Set3 HDAC complex.

## Results

### Identification of suppressor mutations of *pkr*

In a previous study, 67 spontaneous suppressors of the *pkr* mutant were isolated from different culture plates and categorized into three groups based on their growth rate, including 12 of them with suppressor mutations in the *CPK1* gene [22]. To identify suppressor mutations in the remaining suppressor strains that had no mutations in *CPK1*, 10 of them with various growth rates and colony morphology were selected for whole genome sequencing (WGS) analysis (S1 and S2 Figs) [22,30]. Mutations were identified by comparative analysis with the original *pkr* mutant used for suppressor isolation and updated PH-1 genome sequence [31] in 13 predicted genes in *F*. *graminearum* (Table 1). Two frameshift mutations that were caused by an insertion of T after C^6065^ and deletion of 7 base pairs (C^6595^GCTACC^6601^) in FGRRES_00324 [17,32], an ortholog of yeast *SNT1*, were identified in two suppressor strains H11 and H17 (Table 1). Frameshift mutations in FGRRES_16648, an ortholog of yeast *BLM10* also were identified in two suppressor strains. For the other genes, including orthologs of yeast *PRE5*, *PRE6*, *CYC8*, *MIG1*, *NHP6*, and *SGF73*, suppressor mutations were only identified in one suppressor strain each (Table 1). Interestingly, suppressor H11 had frameshift mutations in both *FgSNT1* and *FgBLM10*.

**Table 1:** Mutations identified in the ORFs of predicted genes in suppressors of *pkr*.

Suppressor Strain	Predicted gene	Yeast ortholog	Mutations*	
			DNA	Protein
**Mutations identified by whole genome sequencing analysis**				
H4	FGRRES_07282	*PRE6*	G^410^ to A	D82N
H6	FGRRES_05222	*PRE5*	A^325^ to G	K62E
H10	FGRRES_16648	*BLM10*	ΔT^1220^-C^3362^	S392fs
H11	FGRRES_16648	*BLM10*	ΔG^4185^	G1380fs
	FGRRES_00324	*SNT1*	Insertion of a T after C^6065^	A1958fs
H17	FGRRES_00324	*SNT1*	ΔC^6595^GCTACC^6601^	Y2135fs
H22	FGRRES_12901	*CYC8*	G^314^ to A	G88D
	FGRRES_10588	No homolog	ORF deletion	Del
	FGRRES_10589	No homolog	ORF deletion	Del
	FGRRES_00887	No homolog	ORF deletion	Del
H28	FGRRES_03942	No homolog	G^467^ to T	R140M
H30	FGRRES_00385	NHP6A/6B	T^281^ to C	L57P
	FGRRES_04083	No homolog	Insertion of a T at G^1209^	M368fs
H34	FGRRES_05396	*SGF73*	G^928^ to A	R275K
H57	FGRRES_09715	*MIG1*	A^211^ to G	K71E
**Mutations identified by amplifying and sequencing *FgSNT1***				
H5	FGRRES_00324	*SNT1*	Insertion of a T after C^6065^	A1958fs
H7	FGRRES_00324	*SNT1*	Insertion of a T after C^6065^	A1958fs
H16	FGRRES_00324	*SNT1*	ΔC^6595^GCTACC^6601^	Y2135fs
H26	FGRRES_00324	*SNT1*	ΔC^6595^GCTACC^6601^	Y2135fs
H29	FGRRES_00324	*SNT1*	ΔC^6595^GCTACC^6601^	Y2135fs
H44	FGRRES_00324	*SNT1*	ΔC^6595^GCTACC^6601^	Y2135fs
H51	FGRRES_00324	*SNT1*	ΔC^6595^GCTACC^6601^	Y2135fs
H52	FGRRES_00324	*SNT1*	ΔC^6595^GCTACC^6601^	Y2135fs
H53	FGRRES_00324	*SNT1*	ΔC^6595^GCTACC^6601^	Y2135fs

* Mutations identified in the DNA sequence of predicted genes resulting in changes in amino acid sequences.

fs, frameshift mutant.

Del, deletion of the whole open reading frame

**Fig 1:**
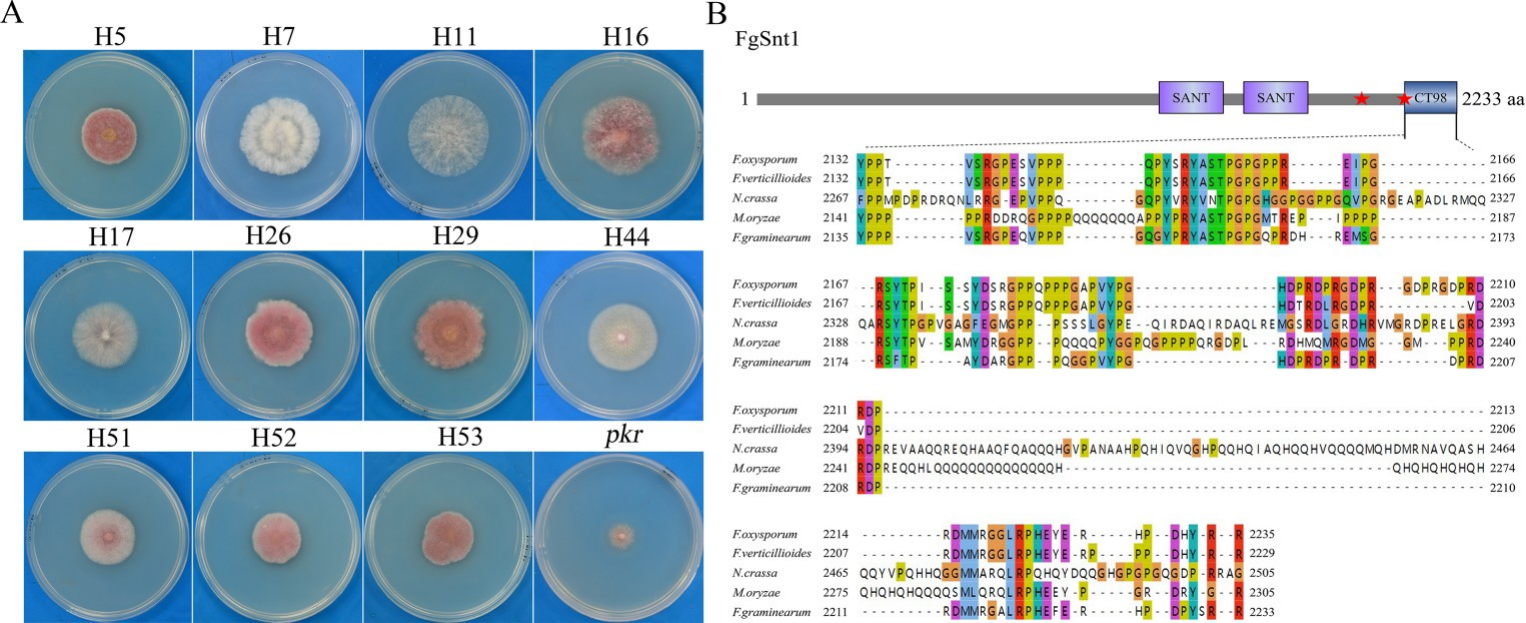
Suppressors of *pkr* with mutations in *FgSNT1* and structural features of FgSnt1. **(A).** Three-day-old PDA cultures of the *pkr* mutant and marked suppressors with mutations in FgSnt1. **(B).** Schematic drawing of the FgSnt1 protein and alignment of its C-terminal 98-aa with orthologs from *Magnaporthe oryzae* (Mo), *Neurospora crassa* (Nc), *F*. *oxysporum* (Fo), and *F*. *verticillioides* (Fv). Stars mark the sites of suppressor mutations. SANT, SANT domain; CT98, the C-terminal 98-aa of FgSnt1.

Snt1 is a core component of the Set3 HDAC complex that is well conserved from yeast to human and important for H3 and H4 acetylation [10,33,34]. Because the relationship between PKA signaling and Set3 HDAC is not well characterized, we selected *FgSNT1* for further characterization in this study. First, we amplified and sequenced the *FgSNT1* gene in the remaining suppressor strains and found nine of them had frameshift mutations (Table 1). All 11 spontaneous suppressor strains with mutations in *FgSNT1* grew faster than the *pkr* mutant but they varied in growth rate and colony morphology (Fig 1A), which may be caused by additional mutations in the genome, such as the frameshift mutation in *FgBLM10* in suppressor H11. When orthologs of yeast *BLM10*, *PRE5*, *PRE6*, *CYC8*, *MIG1*, and *NHP6* were amplified and sequenced, suppressor H5 was found to have a non-sense mutation in *FgBLM10* (S1 Table). For example, suppressors H5, H7, and H11 had the same insertion of a T after C^6065^ that will result in a frameshift and truncation of the C-terminal 275 amino acids (CT275, residues 1958–2233) but they differed in mutations in *FgBLM10*. Whereas H7 had no and H11 had a suppressor mutation, suppressor H5 had a nonsense mutation in *FgBLM10* when it was amplified and sequenced (S1 Table). All other 8 suppressors had the same deletion of 7 base pairs (C^6595^GCTACC^6601^) that will result a frameshift and truncation of the C-terminal 98 amino acids (CT98, residues 2135–2233). FgSnt1 has two conserved SANT DNA-binding domains in the middle region (Fig 1B) that are not affected by these suppressor mutations. In comparison with its orthologs from other fungi, the C-terminal 98 residues of FgSnt1 were conserved in Sordariomycetes, including *Neurospora crassa* and *M*. *oryzae* and this region is proline-rich and glycine-rich (Fig 1B).

### Deletion of the C-terminal region of FgSnt1 rescues the defects of the *pkr* mutant

To verify the suppressive effects of truncations in *FgSNT1* on the *pkr* mutant, we used the split marker approach to generate the *FgSNT1*^ΔCT98^ gene replacement construct in which the C-terminal 98 amino acids were replaced with the geneticin-resistance cassette (S3 Fig). Three *FgSNT1*^ΔCT98^ *pkr* mutant strains (Table 2) that were resistant to both geneticin and hygromycin were identified. They had identical phenotypes although only data for one of them, mutant SCP2, were presented.

**Table 2:** The wild-type and mutant strains of *F*. *graminearum* used in this study.

Strains	Brief description	References
PH-1	Wild type	[6]
P1	*pkr* deletion mutant of PH-1	[22]
C1M-1	*cpk1* deletion mutant of PH-1	[23]
H1 to H67	Spontaneous suppressors of *pkr* mutant	[22]
S1	*Fgsnt1* deletion mutant of PH-1	This study
S7	*Fgsnt1* deletion mutant of PH-1	This study
S10	*Fgsnt1* deletion mutant of PH-1	This study
SC3	*FgSNT1*^ΔCT98^ mutant of PH-1	This study
SC7	*FgSNT1*^ΔCT98^ mutant of PH-1	This study
SC11	*FgSNT1*^ΔCT98^ mutant of PH-1	This study
SP4	*Fgsnt1 pkr* mutant	This study
SP7	*Fgsnt1 pkr* mutant	This study
SP10	*Fgsnt1 pkr* mutant	This study
SP13	*Fgsnt1 pkr* mutant	This study
SCP2	*FgSNT1*^ΔCT98^ *pkr* mutant	This study
SCP5	*FgSNT1*^ΔCT98^ *pkr* mutant	This study
SCP12	*FgSNT1*^ΔCT98^ *pkr* mutant	This study
SDP1	*FgSNT1*^S443D^ transformant of *Fgsnt1 pkr*	This study
SDP2	*FgSNT1*^S443D^ transformant of *Fgsnt1 pkr*	This study
SD1	*FgSNT1*^S443D^ transformant of PH-1	This study
SD3	*FgSNT1*^S443D^ transformant of PH-1	This study

**Fig 2:**
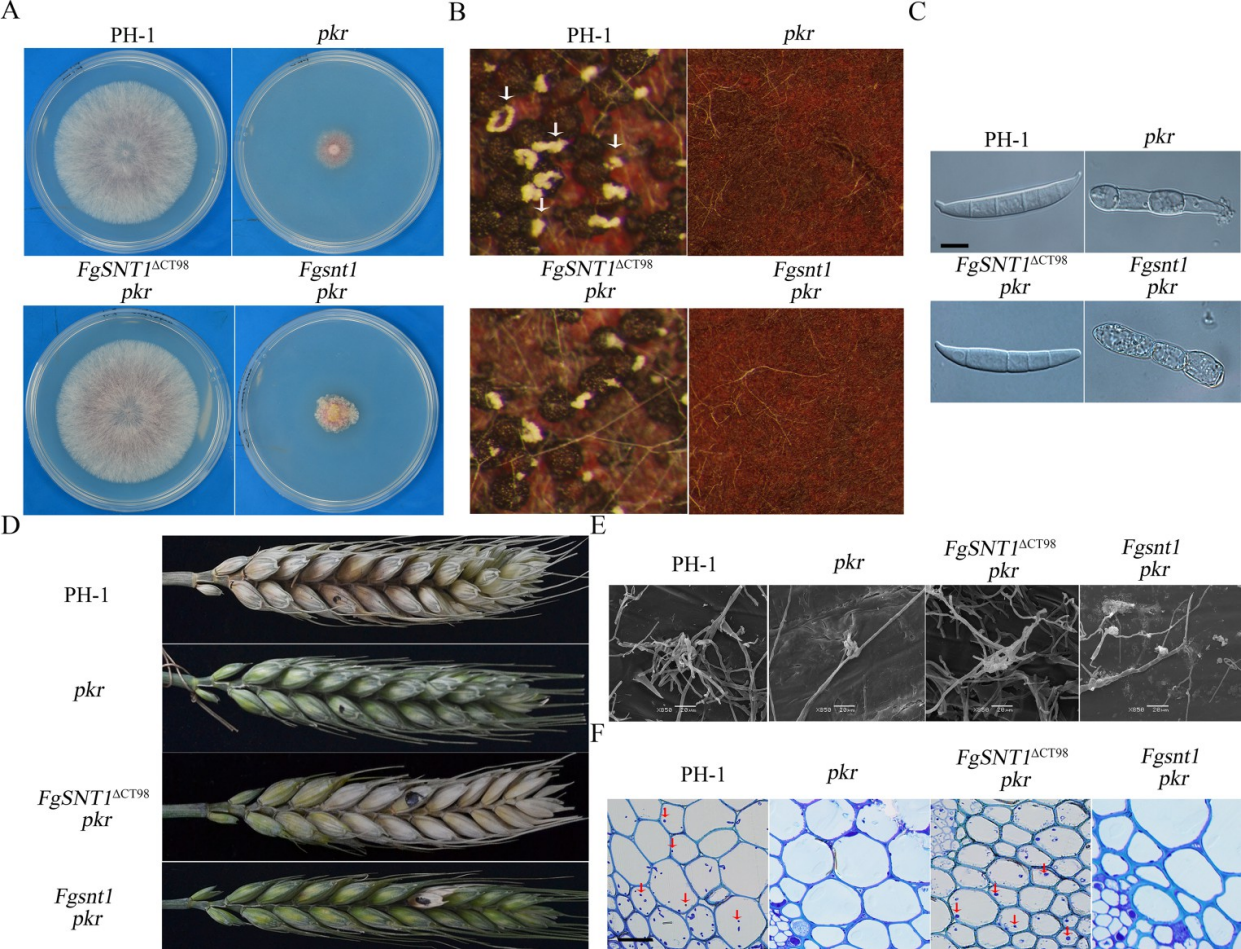
Deletion of the C-terminal region of FgSnt1 partially rescues the defects of *pkr*. **(A).** Three-day-old PDA cultures of the wild-type strain PH-1 and the *pkr*, *FgSNT1*^ΔCT98^ *pkr* and *Fgsnt1 pkr* mutants. **(B).** Perithecia from mating cultures of the same set of strains were examined at 12 days post-fertilization (dpf). Arrows point to ascospore cirrhi oozed out from black perithecia **(C).** Conidia of the same set of strains from 5-day-old CMC culture. Bar = 10 μm. **(D).** Wheat heads inoculated with the marked strains were examined for head blight symptoms at 14 days post-inoculation (dpi). Black dots mark the inoculated spikelets. **(E).** Infection cushions formed on wheat lemma were examined by SEM under ×850 at 2 dpi. Scale bars = 20 μm. **(F).** Thick sections of wheat rachises inoculated with the marked strains were examined for invasive hyphae at 5 dpi.

In comparison with the *pkr* mutant, the *FgSNT1*^ΔCT98^ *pkr* mutant grew faster (Fig 2A) and produced fertile perithecia (Fig 2B). It also produced conidia with normal morphology (Fig 2C) and was normal in conidiation (Table 3). In infection assays with flowering wheat heads, the *FgSNT1*^ΔCT98^ *pkr* mutant caused typical head blight symptoms (Fig 2D) and had a disease index of 7.7 (Table 3). The *FgSNT1*^ΔCT98^ *pkr* mutant was able to form infection cushions on wheat lemma (Fig 2E) and spread to nearby spikelets (Fig 2F). These results indicate that truncation of the C-terminal region of FgSnt1 partially rescues the defects of the *pkr* mutant in growth, conidiation, and pathogenesis.

**Table 3:** Phenotypes of the *Fgsnt1* mutant and its transformant strains in growth, conidiation, and plant infection.

Strain	Growth rate(mm/day)^a^^,^^b^	Conidiation (×10^4^ conidia/ml)^a^^,^^c^	Disease Index^a^^,^^d^
PH-1	10.5 ± 0.1^**a**^	235.8 ± 28.9^**a**^	10.9 ± 3.0^**a**^
*pkr*	2.3 ± 0.2^c^	7.5 ± 2.1^e^	0.0 ± 0.0^d^
*Fgsnt1*	10.0 ± 0.2^**a**^	76.3 ± 12.3^c^^d^	6.2 ± 1.1^c^
*FgSNT1* ^ΔCT98^	10.1 ± 0.1^**a**^	195.8 ± 26.5^b^	8.4 ± 2.8^b^
*Fgsnt1 pkr*	2.0 ± 0.4^c^	65.0 ± 16.0^d^	1.0 ± 0.0^d^
*FgSNT1*^ΔCT98^ *pkr*	10.0 ± 0.2^**a**^	192.5 ± 24.0^b^	7.7 ± 3.6^b^^c^
*FgSNT1*^S443D^ *pkr*	4.6 ± 0.2^b^	90.0 ± 14.3^c^	6.3 ± 1.0^c^

^**a**^ Standard deviation (mean ± standard deviation) were calculated from at least three independent measurements. Different letters indicate significant differences based on ANOVA analysis followed by Duncan’s multiple range test (P = 0.05) in a, b, c, d, e.

^**b**^ Average daily extension of colony radium.

^**c**^ Conidiation was measured with 5-day-old Carboxymethylcellulose(CMC) culture.

^**d**^ Diseased spikelets per wheat head examined 14 dpi.

To determine whether deletion of the entire *FgSNT1* can rescue the *pkr* mutant, we also used the gene replacement approach to generate the *Fgsnt1 pkr* double mutant. All four *Fgsnt1 pkr* mutant strains (Table 2) were similar to the *pkr* mutant in growth rate, conidiation, sexual reproduction, and virulence although only data for strain SP4 were presented (Fig 2). Therefore, truncation of its C-terminal region, but not deletion of the entire *FgSNT1*, is suppressive to the *PKR* deletion.

### The C-terminal region of FgSnt1 is not essential for its functions in conidiation and plant infection

**Fig 3:**
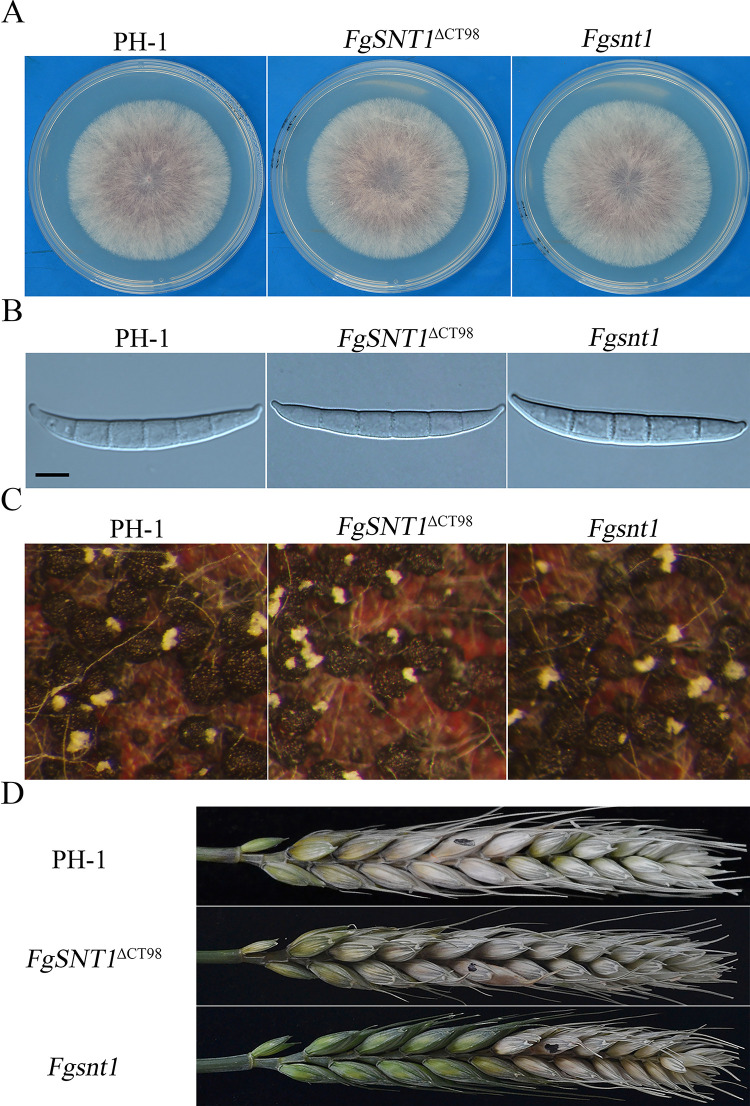
Assays for the phenotypes of the *Fgsnt1* and *FgSNT1*^ΔCT98^ mutants. **(A).** Three-day-old PDA cultures of the wild type (PH-1) and the *Fgsnt1* and *FgSNT1*^ΔCT98^ mutants. **(B).** Conidia from 5-day-old CMC cultures were examined for morphological defects. Bar = 10 μm. **(C).** Perithecia from mating cultures of the marked strains were examined at 12 dpf. **(D).** Wheat heads inoculated with the marked strains were examined for head blight symptoms at 14 dpi. Black dots mark the inoculated spikelets.

To determine the functions of *FgSNT1* and its C-terminal region, we then transformed the *FgSNT1* and *FgSNT1*^ΔCT98^ gene replacement constructs into the wild-type strain PH-1, respectively. The resulting *Fgsnt1* and *FgSNT1*^ΔCT98^ gene replacement mutants had no significant changes in vegetative growth (Fig 3A, Table 3), conidium morphology (Fig 3B), and sexual reproduction (Fig 3C). However, conidiation was significantly reduced in the *Fgsnt1* deletion mutant in comparison with the wild type (Table 3). For the *FgSNT1*^ΔCT98^ mutant, it was only slightly reduced (less than 20% reduction) in conidiation compared to PH-1 but it produced over two-fold more conidia than the *Fgsnt1* mutant (Table 3).

In infection assays with wheat heads, the *Fgsnt1* and *FgSNT1*^ΔCT98^ deletion mutants were still pathogenic and caused typical head blight symptoms on inoculated spikelets (Fig 3D). However, both of them were reduced in virulence in comparison with PH-1. On average, the disease index was 10.9, 6.2, and 8.4, respectively, for the wild type, *Fgsnt1*, and *FgSNT1*^ΔCT98^ strains (Table 3). Therefore, the *Fgsnt1* and *FgSNT1*^ΔCT98^ deletion mutants had approximately 43% and 23% reduction in virulence. These results indicate that *FgSNT1* is important for conidiation and plant infection. Because deletion of CT98 had much less effects on conidiation and virulence than deletion of the entire gene, the C-terminal region of FgSnt1 likely has a minor role in conidiation and pathogenicity but is not essential for its normal functions in *F*. *graminearum*.

### CT98 of FgSnt1 negatively regulates histone H4 acetylation in the *pkr* mutant

**Fig 4:**
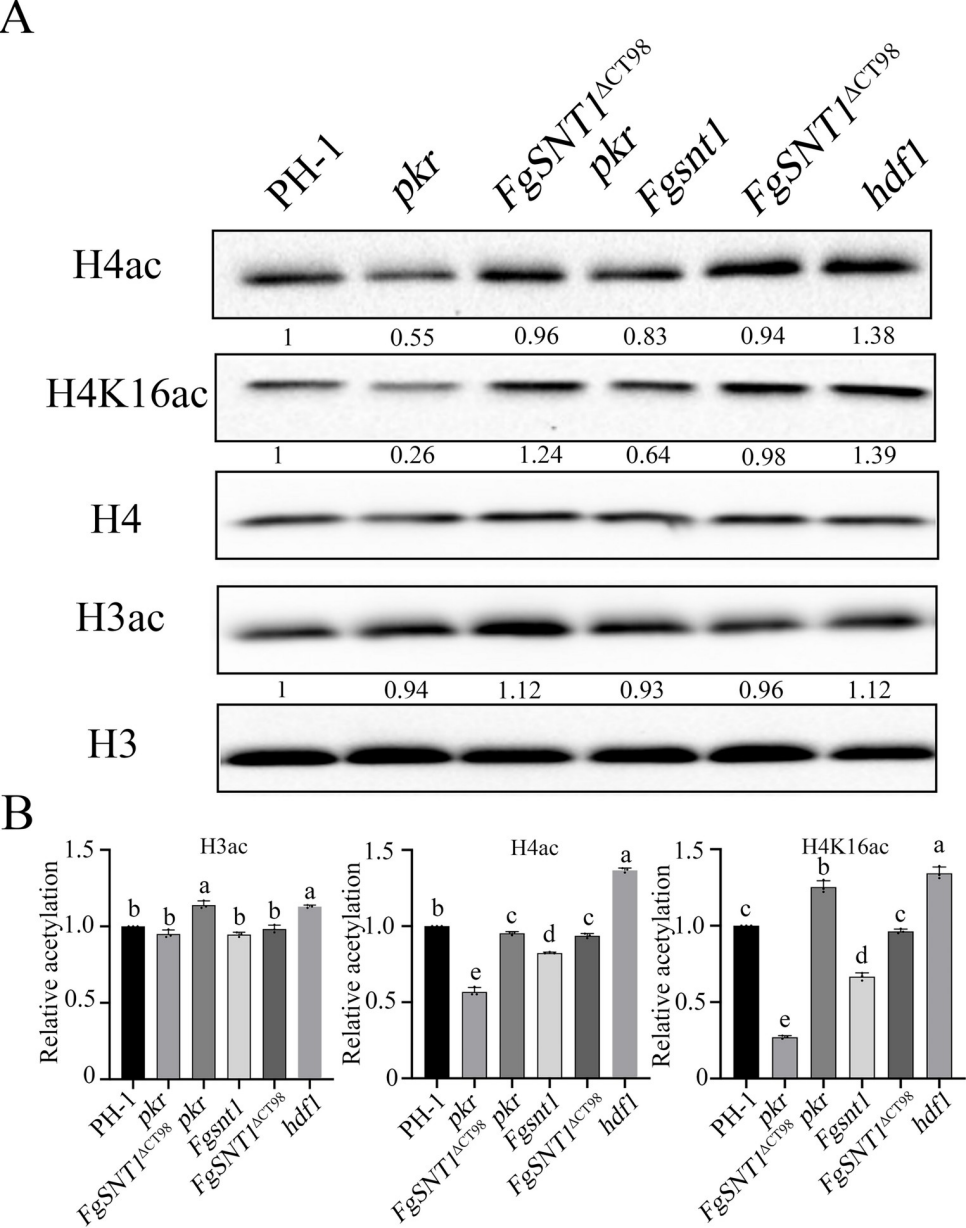
Deletion of the C-terminal region of FgSnt1 increases H4 acetylation in the *pkr* mutant. **(A).** Western blots of total proteins isolated from PH-1 and the *pkr*, *FgSNT1*^ΔCT98^ *pkr*, *Fgsnt1*, *FgSNT1*^ΔCT98^, and *hdf1* mutants were detected with antibodies specific for H3ac, H4ac, and H4K16ac. Detection with anti-H3 and anti-H4 antibodies was used as a loading control. **(B).** Quantitative analysis of the histone acetylation level by calculating the ratio of the quantified acetylated and non-acetylated H3 and H4 proteins.

Because yeast Snt1 is a conserved component of the Set3 HDAC complex, we then assayed global histone acetylation levels with selected anti-H3ac and anti-H4ac antibodies by western blot analysis (Fig 4A). In comparison with the wild type, the *pkr* mutants were significantly reduced in H4 acetylation, but had no significant differences in H3 acetylation compare to the wild type (Fig 4B) (S4A and S4B Figs). The *FgSNT1*^ΔCT98^ and *Fgsnt1* mutants had similar H3 acetylation levels but were slightly reduced in H4 acetylation compared to the wild-type strain (Fig 4A). The *FgSNT1*^ΔCT98^ *pkr* double mutant had significantly higher H4 acetylation level than the *pkr* mutant although it was still slightly reduced in comparison with PH-1 (Fig 4). However, the *Fgsnt1 pkr* double mutant had a similar H4 acetylation level with the *pkr* mutant (S4C Fig). These results indicate that deletion of CT98, but not the entire *FgSNT1*, partially recovered the reduction of the *pkr* mutant in H4 acetylation.

In yeast, Hos2, a HDAC in the Set3 complex and an ortholog of *F*. *graminearum* Hdf1, is important for H4K16 deacetylation [35]. In comparison with the wild type, the levels of H4 and H4K16 acetylation were increased 38% and 39%, respectively, in the *hdf1* mutant (Fig 4). In contrast, the *pkr* deletion mutant was reduced over 74% in H4K16 acetylation compared to PH-1. The *FgSNT1*^ΔCT98^ *pkr* mutants had a higher level of H4K16 acetylation than PH-1 or *pkr* but lower than the *hdf1* mutant (Fig 4). These results showed that the defect of the *pkr* mutant in H4 acetylation is suppressed by deletion of the C-terminal region of FgSnt1. Because of the conserved role of Pkr regulatory subunit of PKA in cAMP signaling, it is likely that the Set3 HDAC complex is regulated by the cAMP-PKA pathway via FgSnt1 in *F*. *graminearum*. In the absence of *PKR*, the C-terminal region of FgSnt1 may be suppressive to H4 acetylation by interfering with the Hdf1 HDAC activity. Deletion of the C-terminal 98 residues may bypass the requirement of normal cAMP-PKA signaling for regulating activities of the Set3 HDAC complex and the expression of subsets of genes affected by H4 acetylation.

### The C-terminal region of FgSnt1 plays a negative role in its interaction with Hdf1

**Fig 5:**
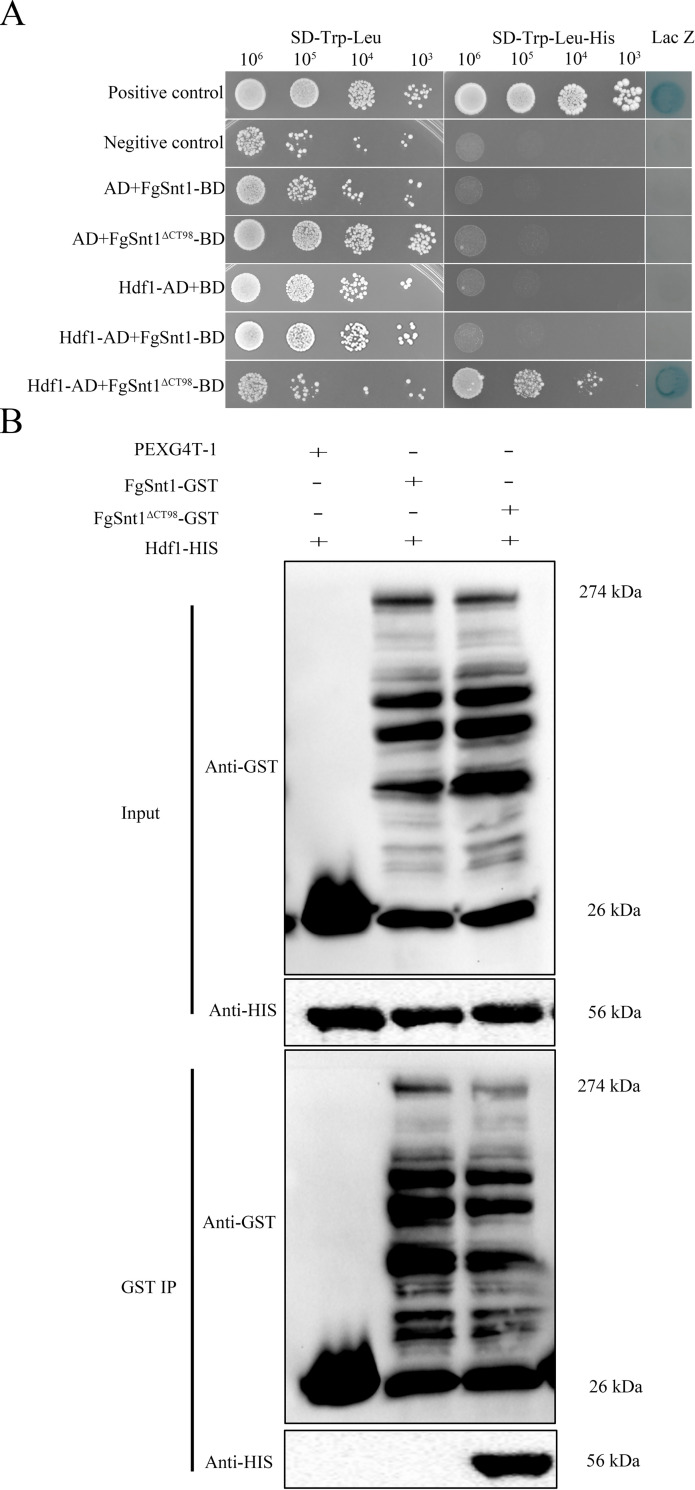
Assays for the interaction of Hdf1 with FgSnt1 and FgSnt1^ΔCT98^. **(A).** Yeast two-hybrid assays for the interaction of Hdf1 (prey) with FgSnt1 or FgSnt1^ΔCT98^ (bait). The positive and negative controls were from the Matchmaker kit. **(B).** Immunoprecipitation (IP) assays for the interaction between Hdf1 with FgSnt1^ΔCT98^ and FgSnt1. Western blots of proteins isolated from *E*. *coli* cells expressing Hdf1-HIS together with FgSnt1-GST or FgSnt1^ΔCT98^-GST (Input) or proteins eluted from anti-GST beads (GST IP) were detected with the marked anti-GST and anti-HIS antibodies. Proteins from *E*. *coli* cells expressing the empty GST vector pGEX4T-1 were used as the control.

To determine the interaction of FgSnt1 with Hdf1 by yeast two-hybrid assays, we generated the bait constructs with FgSnt1 and FgSnt1^ΔCT98^ and co-transformed them with the prey constructs of Hdf1 into yeast strains AH109. Yeast transformants expressing the Hdf1 prey and FgSnt1^ΔCT98^ bait, but not FgSnt1 bait, constructs were able to grow on SD-Trp-Leu-His (synthetic defined (SD) medium without Trp, Leu, His) medium and had LacZ activity (Fig 5A). These yeast two-hybrid assay results suggested that Hdf1 directly interacts with the FgSnt1^ΔCT98^ but not with full-length FgSnt1, at least in yeast. It is likely that CT98 functions as a negative regulator of the FgSnt1-Hdf1 interaction, which may be stimulatory to Hdf1 HDAC activity.

To confirm the importance of FgSnt1^CT98^ in its interaction with Hdf1, we isolated the FgSnt1-GST, FgSnt1^ΔCT98^-GST, and Hdf1-HIS recombinant proteins expressed in *Escherichia coli*. For GST pull down assays, Hdf1-HIS proteins were mixed with FgSnt1-GST or FgSnt1^ΔCT98^-GST proteins and anti-GST agarose beads. On western blots with proteins eluted from anti-GST agarose beads, the Hdf1-HIS band of expected size was detected by an anti-HIS antibody only when Hdf1-HIS proteins were co-incubated with FgSnt1^ΔCT98^-GST but not with FgSnt1-GST proteins (Fig 5B). These results from in vitro assays further suggest an important role of CT98 in the interaction of FgSnt1 with Hdf1.

### Pkr is important for protecting Cpk1 from degradation by 26S proteosome

**Fig 6:**
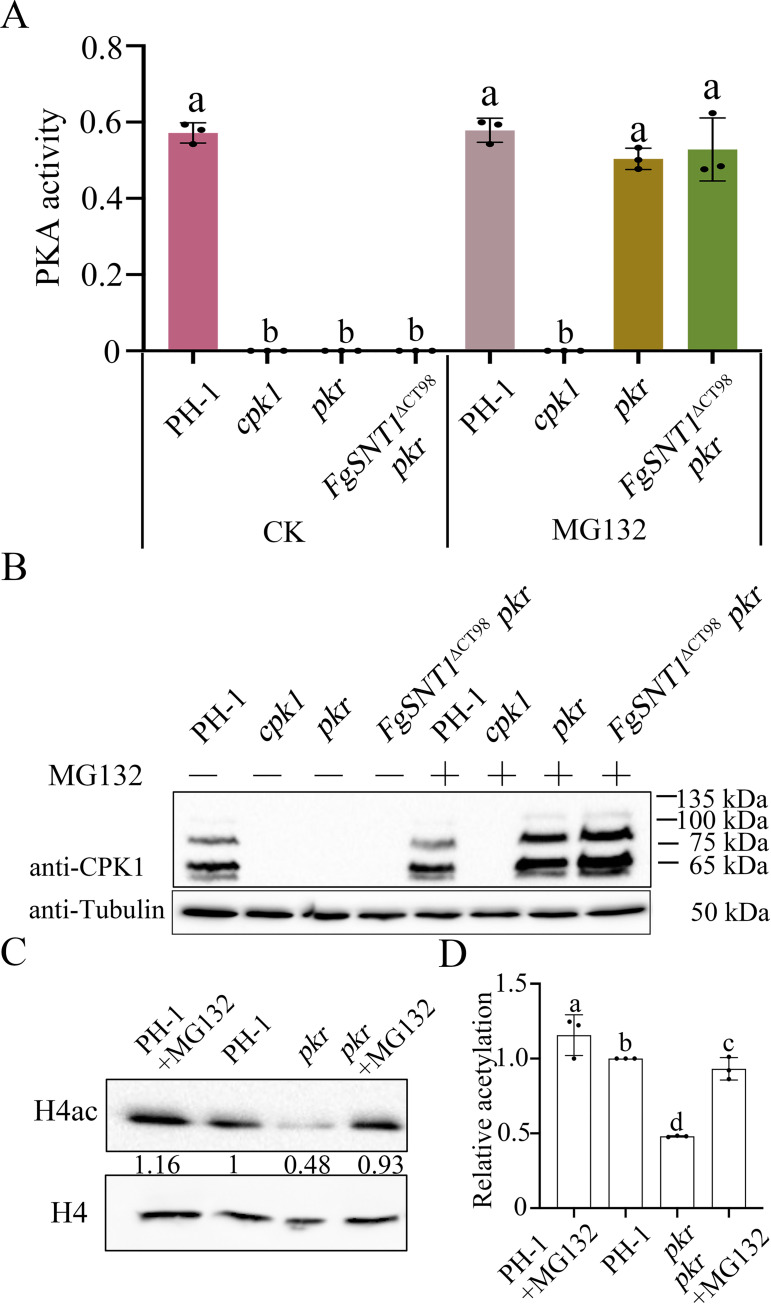
Assays for PKA activities and Cpk1 expression. **(A).** PKA activity was assayed with proteins isolated from hyphae of PH-1, *cpk1* and *pkr* mutant, and *FgSNT1*^ΔCT98^ *pkr* mutant without or with 50 μM MG132 treatment. **(B).** Western blots of total proteins isolated from the marked strains and MG132 treatment were detected with an anti-Cpk1 antibody. The expected size of Cpk1 is 67 kDa. Detection with an anti-tubulin antibody was used as the loading control. **(C).** Western blots of total proteins isolated from PH-1 and the *pkr*, PH-1 treatment with MG132, and *pkr* treatment with MG132 mutants were detected with antibodies for H4ac. Detection with anti-H4 antibodies was used as a loading control. **(D).** Quantitative analysis of the histone acetylation level by calculating the ratio of the quantified acetylated and non-acetylated H4 proteins.

To determine the effects of truncation mutations in *FgSNT1* on PKA activities in the *pkr* mutant, total proteins were isolated from PH-1, *cpk1*, *pkr*, and *FgSNT1*^ΔCT98^ *pkr* and assayed for PKA activities [36]. In comparison with the wild type, the *cpk1* mutant had no detectable PKA activities (Fig 6A). To our surprise, the *pkr* and *FgSNT1*^ΔCT98^ *pkr* mutants also lacked detectable PKA activities (Fig 6A). Because *CPK1* expression was found to be significantly upregulated in the *pkr* mutant in a previous study [22], it is likely that the catalytic subunits of PKA are unstable or degraded in the absence of Pkr regulatory subunits. To test this hypothesis, we generated an anti-Cpk1 antibody with the synthetic oligopeptide VKAGAGDASQFDRYPE. In proteins isolated from vegetative hyphae, the resulting antibody detected a 67-kDa band of expected Cpk1 size and two minor bands in PH-1 but not in the *cpk1* mutant (Fig 6B), indicating that this anti-Cpk1 antibody is suitable for Cpk1 detection. The two minor bands may be related to post-translational modifications of Cpk1 proteins such as ubiquitination. In the *pkr* mutant and *FgSNT1*^ΔCT98^ *pkr* mutant, the 67-kDa Cpk1 band as well as the two minor bands were not detected with the anti-Cpk1 antibody (Fig 6A, 6B), indicating that Pkr is necessary for the stability of Cpk1.

Because suppressor mutations were identified in orthologs of yeast *BLM10*, *PRE5*, and *PRE6* that encode key components of the 26S proteasome (Table 1), we then assayed the effects of MG132, an 26S proteasome inhibitor, on Cpk1 expression. When treated with 50 μM MG132, the Cpk1 band and PKA activities were detected in the *pkr* and *FgSNT1*^ΔCT98^ *pkr* mutants (Fig 6A and 6B), indicating that Cpk1 proteins may be degraded by the 26S proteasome in the *pkr* mutant. In addition, the suppressor H10 with frameshift mutation on FgBlm10 also rescued the Cpk1 stability and PKA activities (S5 Fig). These results indicate that, in the absence of Pkr that is necessary to form stable PKA holoenzymes, Cpk1 proteins are degraded in the *pkr* mutant, which can be suppressed by inhibition of 26S proteasome and genetic mutations in its key components. H4 acetylation of the *pkr* mutant was significantly reduced. When treated with 50 μM MG132, the H4 acetylation of *pkr* partially rescued, and showed reduced significant differences compare to the wild type (Fig 6C and 6D), indicating that the inhibition of Cpk1 degradation rescues the reduction in H4 acetylation of *pkr* mutant.

### FgSnt1 interacts with Cpk1 but not Pkr

**Fig 7:**
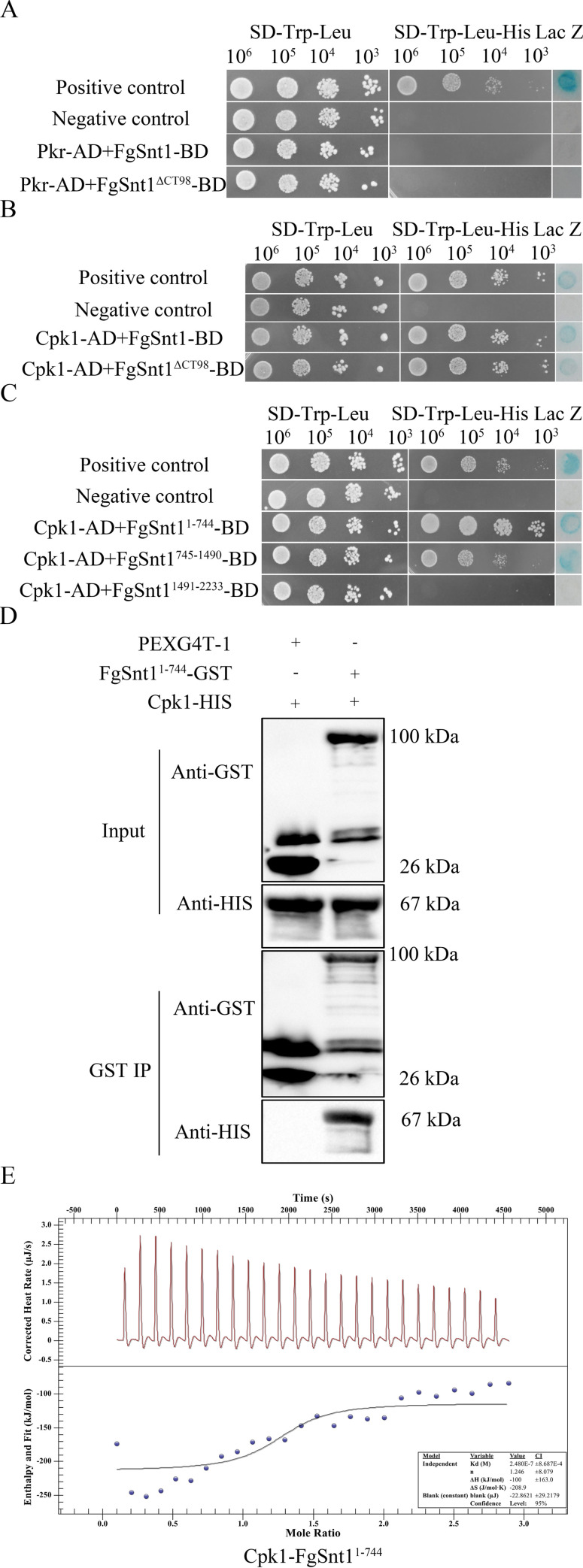
Assays for the interaction of Cpk1 with FgSnt1. **(A).** Yeast two-hybrid assays for the interaction of Pkr (prey) with FgSnt1 or FgSnt1^ΔCT98^ (bait). The positive and negative controls were from the Matchmaker kit. **(B).** Yeast two-hybrid assays for the interaction of Cpk1 (prey) with FgSnt1 or FgSnt1^ΔCT98^ (bait). **(C).** Yeast two-hybrid assays for the interaction of Cpk1with FgSnt1^1-744^, FgSnt1^745-1490^, or FgSnt1^1491-2233^. **(D).** Immunoprecipitation (IP) assays for the interaction between Cpk1 with FgSnt1^1-744^. Western blots of proteins isolated from *E*. *coli* cells expressing Cpk1-HIS together with FgSnt1^1-744^-GST (Input) or proteins eluted from anti-GST beads (GST IP) were detected with the marked anti-GST and anti-HIS antibodies. Proteins from *E*. *coli* cells expressing the empty GST vector pGEX4T-1 were used as the control. **(E).** The ITC thermogram of binding interactions between Cpk1 and FgSnt1^1-744^. The upper panel show raw data obtained from 10 μl injections of Cpk1 to FgSnt1^1-744^. The lower panel display plots of integrated total energy exchanged (as kcal/mol of injected compound) as a function of molar ratio of Cpk1 to FgSnt1^1-744^(dots). Solid lines indicate the fit to a single-site saturation model.

To explore the interaction between PKA and FgSnt1, we generated the bait constructs of FgSnt1 and FgSnt1^ΔCT98^ and co-transformed them with the prey constructs of Pkr or Cpk1. In yeast two-hybrid assays, Pkr had no direct interaction with FgSnt1^ΔCT98^ or full-length FgSnt1 (Figs 7A and S6). However, both FgSnt1 and FgSnt1^ΔCT98^ interacted with Cpk1 in yeast two-hybrid assays (Figs 7B and S6).

To determine which region of FgSnt1 interacts with Cpk1, we generated bait constructs of FgSnt1 with the N-terminal 1–744 aa, middle 745–1490 aa, and C-terminal 1491–2233 aa. In yeast two-hybrid assays, FgSnt1^1-744^ and FgSnt1^745-1490^ but not FgSnt1^1491-2233^ interacted with Cpk1 (Fig 7C). FgSnt1^1-744^ appeared to have a stronger interaction with Cpk1 than FgSnt1^745-1490^ (Figs 7C and S6).

To confirm their interactions, we detected the interaction of FgSnt1^1-744^-GST and Cpk1-HIS with GST pull down assays. The in vitro result showed that the Cpk1-HIS band of expected size was detected by an anti-HIS antibody in samples mixing with FgSnt1^1-744^-GST (Fig 7D). The isothermal titration calorimetry (ITC) assay [37] was also used to measures the amount of heat absorbed or released as Cpk1-HIS is titrated into FgSnt1^1-744^-GST (Figs 7E and S7). The dissociation constant (Kd), stoichiometry of the ligand-to-protein binding (n), enthalpy change (ΔH), and entropy change (ΔS) were determined (Fig 7E). Theoretical fits to FgSnt1^1-744^-Cpk1 association data were obtained using a single binding site model, confirming the interaction of FgSnt1^1-744^-GST and Cpk1-HIS.

### FgSnt1 is a direct phosphorylation target of Cpk1

**Fig 8:**
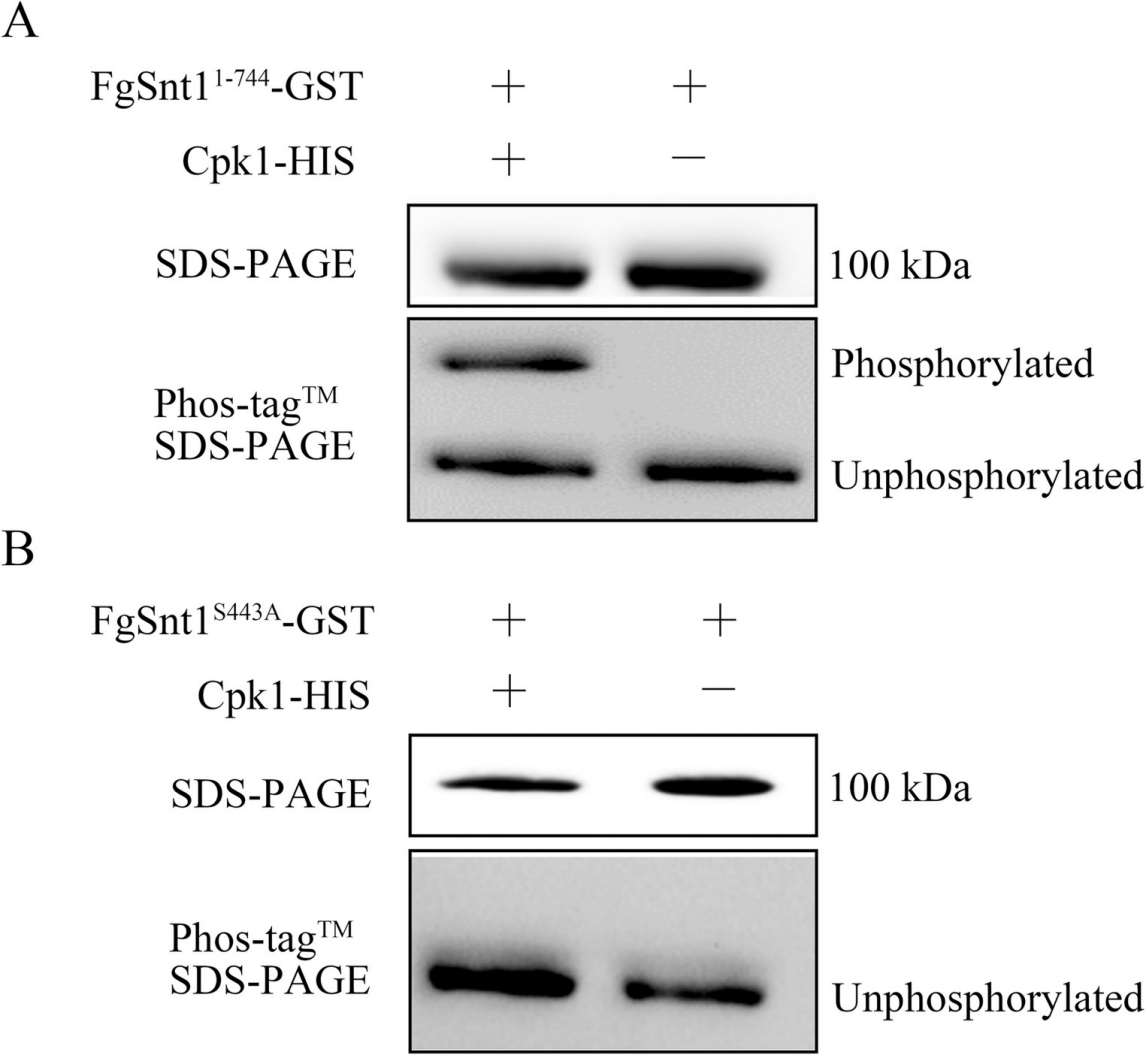
Assays for the phosphorylation of FgSnt1^1-744^ by Cpk1. **(A).** In vitro kinase assays with FgSnt1^1-744^-GST and Cpk1-HIS recombinant proteins. Blots of SDS-PAGE or 20μM Mn^2+^-Phos-tag SDS-PAGE gels were detected with an anti-GST antibody for the FgSnt1^1-744^-GST bands. **(B).** In vitro kinase assays with FgSnt1^S443A^-GST and Cpk1-HIS fusion proteins. The S443 to A mutation in FgSnt1^1-744^-GST eliminated its phosphorylation by Cpk1.

To test whether FgSnt1 is phosphorylated by Cpk1, we conducted *in vitro* phosphorylation assays with purified recombinant FgSnt1^1-744^-GST and Cpk1-HIS proteins [38,39]. On Phos-tag SDS gels, FgSnt1^1-744^-GST proteins incubated with Cpk1 and ATP shifted to a slowly migrating band, suggesting that FgSnt1^1-744^ was phosphorylated by Cpk1 (Fig 8A). In the control without Cpk1, gel mobility shift of the FgSnt1^1-744^-GST band was not observed (Fig 8A).

Primary sequence analysis predicts the presence of five PKA phosphorylation sites in the N-terminal region of FgSnt1 (S18, S38, S322, S443, and S511) (S3 Table), which fit the PKA phosphorylation motif [R][R][X][S/T] (S8 Fig). To test which sites are phosphorylated by Cpk1, we generated the FgSnt1^S18A^-, FgSnt1^S38A^-, FgSnt1^S322A^-, FgSnt1^S443A^-, and FgSnt1^S511A^-GST constructs by replacing the respective serine with alanine [40] in the FgSnt1^1-744^-GST construct, and isolated these recombinant proteins expressed in *E*. *coli*. In the *in vitro* kinase assays, Cpk1 failed to phosphorylate the FgSnt1^S443A^-GST protein (Fig 8B), indicating that S443 is the critical phosphorylate site (Fig 8). However, unlike the FgSnt1^S443A^ -GST protein, the remaining recombinant proteins FgSnt1^S18A^-, FgSnt1^S38A^-, FgSnt1^S322A^-, and FgSnt1^S511A^-GST were still phosphorylated by Cpk1, indicating that phosphorylation of FgSnt1^1-744^ by Cpk1 is not affected by the S18A, S38A, S322A, and S511A mutations (Figs 8B and S9). Therefore, Cpk1 specifically phosphorylated FgSnt1^1-744^ at S443. Sequence alignments showed that only S443 of these phosphorylation sites is conserved among FgSnt1 orthologs from filamentous ascomycetes (S8 Fig), suggesting its phosphorylation by PKA may be also conserved with important function in other ascomycetes.

### S443D mutation in FgSnt1 partially rescues defects of *pkr* mutant

**Fig 9:**
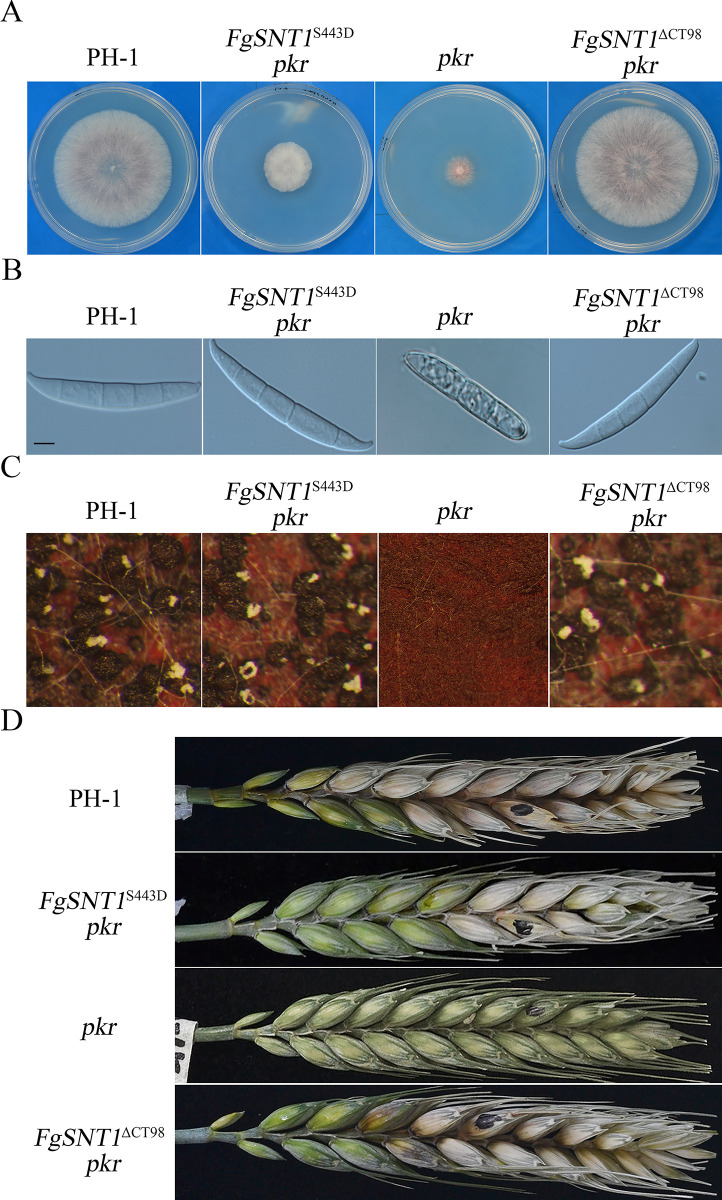
The S443D mutation in *FgSNT1* partially rescues defects of the *pkr* mutant. **(A).** Three-day-old PDA cultures of PH-1 and the *FgSNT1*^S443D^ *pkr*, *FgSNT1*^ΔCT98^ *pkr* and *pkr* mutants. **(B).** Conidia of the same set of strains from 5-day-old CMC culture. Bar = 10 μm. **(C).** Perithecia from mating cultures of the marked strains were examined at 12 dpf. **(D).** Wheat heads inoculated were examined for head blight symptoms at 14 dpi. Black dots mark the inoculated spikelets.

To determine the role of S443 phosphorylation, we introduced an S443D mutation by overlapping PCR and transformed the resulting *FgSNT1*^S443D^ gene replacement construct into the *Fgsnt1 pkr* mutant. Transformants resistant to geneticin and 5-fluorodeoxyuridine were screened by PCR with primers specific for the mutant alleles. The *FgSNT1*^S443D^ *pkr* transformants (Table 2) were further confirmed by sequencing analysis for the S443D mutation. The *FgSNT1*^S443D^ *pkr* transformants grew faster than the *pkr* mutant although it still grew slower than the wild type and *FgSNT1*^ΔCT98^ *pkr* (Fig 9A, Table 3). These results indicate that the S443D mutation in FgSnt1 partially rescued the growth defect of *pkr* mutant. Because the *FgSNT1*^S443D^ *pkr* transformants still grew slower than *FgSNT1*^ΔCT98^ *pkr*, it is likely that other putative phosphorylation sites (Table 3, S9 Fig) may also contribute to the functional relationship between FgSnt1 and Pkr.

In comparison with the *pkr* mutant, conidium morphology was normal (Fig 9B) and conidiation was increased in the *FgSNT1*^S443D^ *pkr* transformant although it still produced fewer conidia than the wild type. On self-mating carrot agar plates, *FgSNT1*^S443D^ *pkr* produced abundant perithecia and ascospore cirrhi (Fig 9C). In infection assays, the *FgSNT1*^S443D^ *pkr* mutant caused typical head blight symptoms on inoculated wheat heads and had a disease index of 6.3, which is similar to that of *FgSNT1*^ΔCT98^ *pkr* and approximately 50% of that of the wild type (Fig 9D). These data indicate that the S443D mutation in FgSnt1 also partially rescued the defect of the *pkr* mutant in plant infection but fully rescued its defect in conidium morphology and sexual reproduction.

### Expressing the *FgSNT1*^S443D^ allele and exogenous cAMP treatment increase H4 acetylation

**Fig 10:**
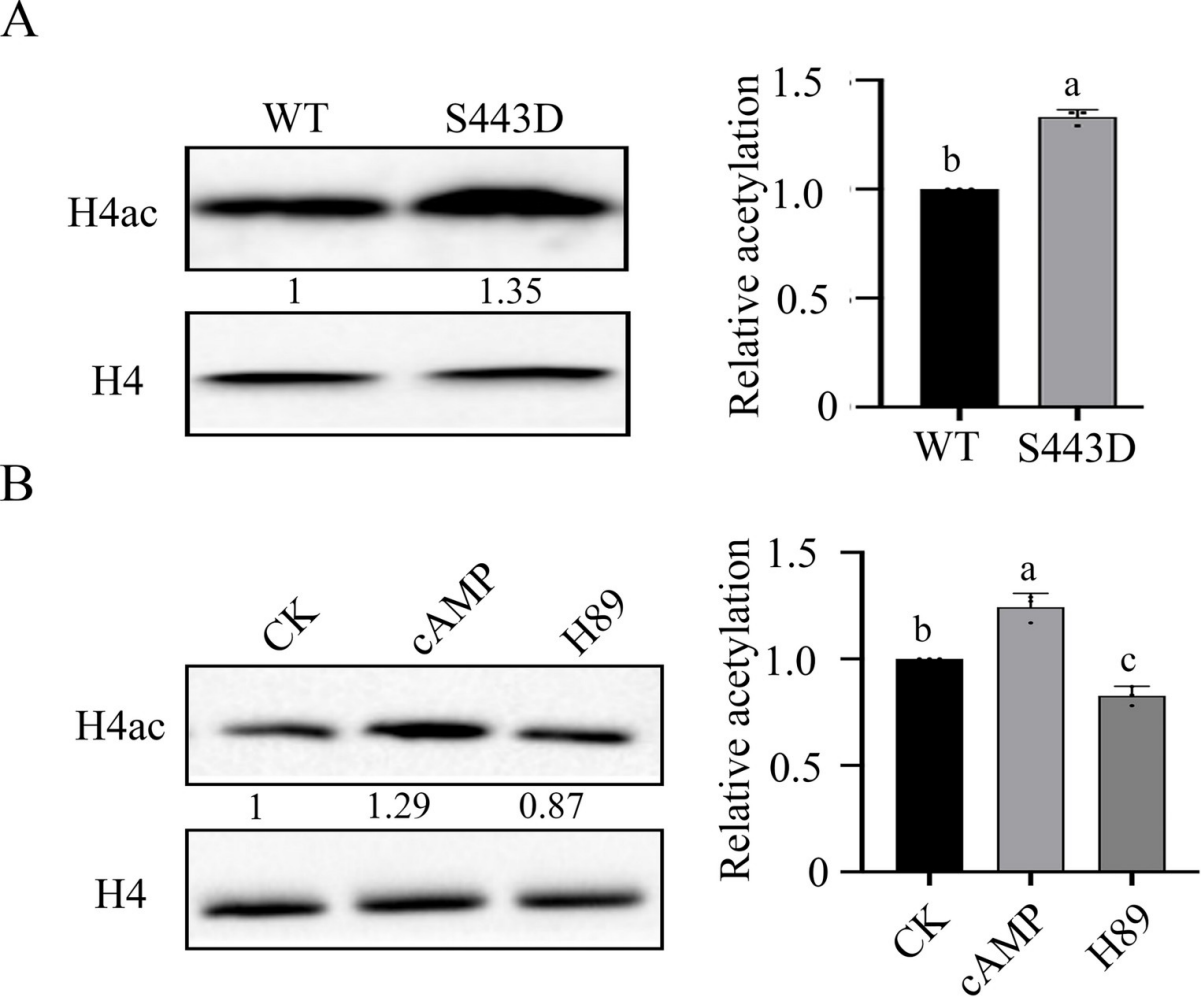
Phosphorylation of FgSnt1 ^S443^ is important for H4 acetylation. **(A).** Western blots of total proteins isolated from PH-1 (WT) and its transformant expressing *FgSNT1*^S443D^ (S443D) were detected with anti-H4 and anti-H4ac antibodies (left) and quantified for the band intensities with Image J to estimate the relative H4 acetylation level after normalization with the non-acetylated H4 band. Data from three replicates were used to estimate the error bar. **(B).** Western blots of proteins isolated from regular PH-1 cultures (CK) and cultures treated with 50 μM cAMP (cAMP) or 50 μM H89 (H89) were detected with anti-H4 and anti-H4ac antibodies and quantified for the band intensities with Image J to estimate the relative H4 acetylation level after normalization with the non-acetylated H4 band. Data from three replicates were used to estimate the error bar.

Because *FgSNT1*^S443D^ partially rescued the defects of the *pkr* mutant, it is possible that FgSnt1 is phosphorylated by PKA to regulate HDAC activities of Hdf1 or the Set3 complex as a whole. When assayed with the anti-H4ac antibody, the *FgSNT1*^S443D^ transformant had increased H4 acetylation in comparison with the wild type (Fig 10A). These results suggest that expression of the dominant active *FgSNT1*^S443D^ allele may reduce the Set3 or Hdf1 HDAC activities in *F*. *graminearum*.

To further characterize the relationship between PKA activity and H4 acetylation, we treated PH-1 hyphae with 50 μM cAMP and 50 μM H89, a PKA inhibitor [41]. H4 acetylation was significantly increased by exogenous cAMP (Fig 10B). In contrast, treatments with H89 reduced H4 acetylation (Fig 10B). Taken together, overstimulating and inhibiting the cAMP-PKA pathway appear to have opposite effects on H4 acetylation, likely by affecting the phosphorylation of FgSnt1 and Set3/Hdf1 HDAC activities in *F*. *graminearum*.

### The S443D, but not S443A, mutation affects the interaction between the N-terminal and C-terminal regions of FgSnt1

**Fig 11:**
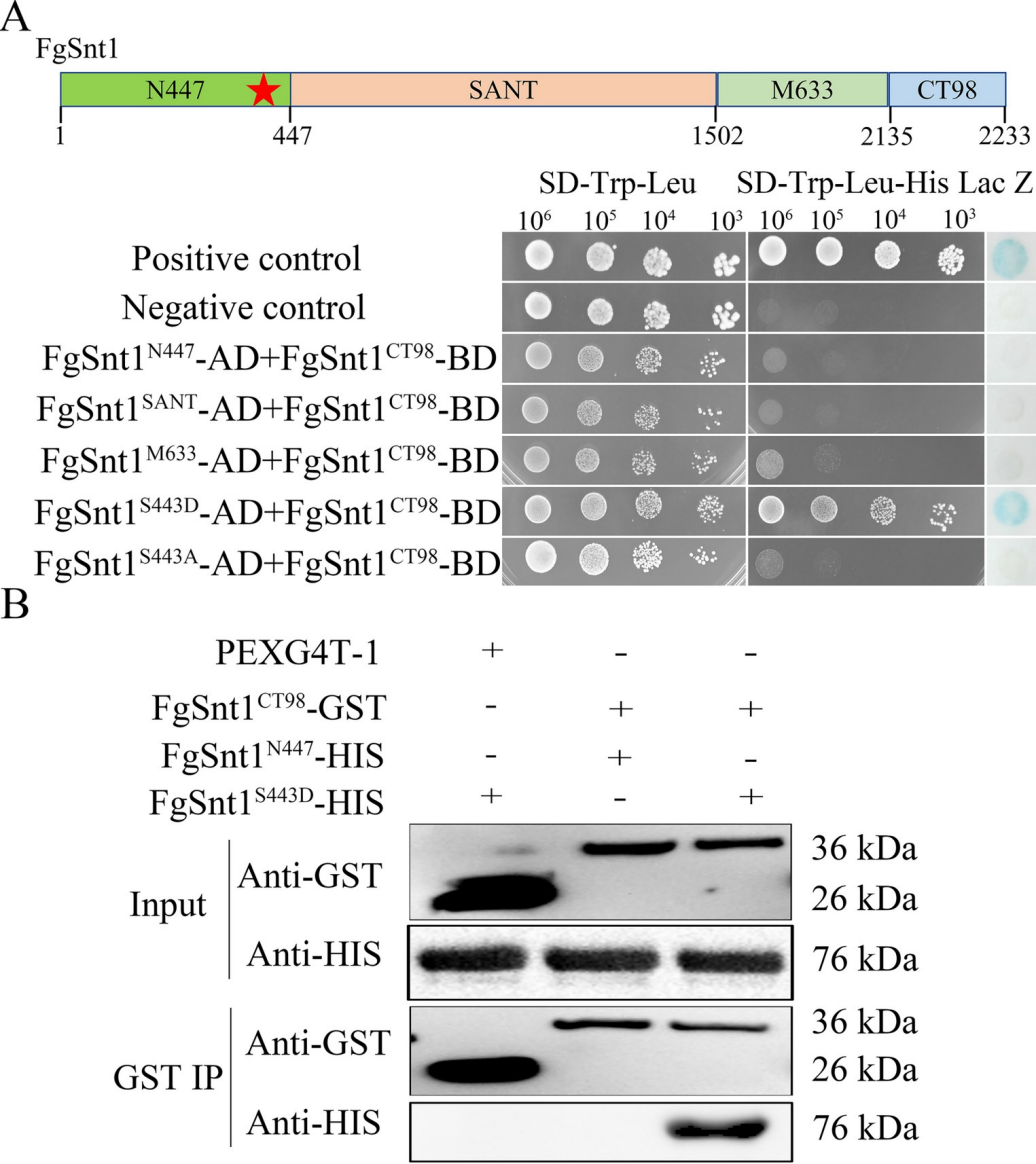
Assays for intramolecular interactions in FgSnt1 and the effect of S443D mutation. **(A).** Yeast two-hybrid assays for the intramolecular interactions in FgSnt1. Yeast cells expressing the marked bait (BD) and prey (AD) constructs were assayed for growth on SD-Trp-Leu (left), SD-Trp-Leu-His plates (middle), and LacZ activities (right). N447, SANT, M633, and CT98 are fragments containing residues 1–447, 448–1502 (SANT domains), 1503–2136, and 2135–2233 of FgSnt1, respectively. FgSnt1^S443D^ and FgSnt1^S443A^ have the S443D and S443A mutations in N447. The positive and negative controls are from the Matchmaker kit. **(B).** IP assays for the interaction of FgSnt1^CT98^ with FgSnt1^N447^ and FgSnt1^S443D^. Blots of the mixtures of marked recombinant proteins (input) and proteins eluted from eluted from anti-GST beads (GST IP) were detected with the anti-GST and anti-HIS antibodies. Proteins from *E*. *coli* cells expressing the empty GST vector pGEX4T-1 were used as the control.

Because deletion of CT98 region and expression of the *FgSNT1*^S443D^ allele both partially rescued the defects of the *pkr* mutant, it is likely that phosphorylation of S443 by PKA has similar effects as deletion of CT98 on the function of FgSnt1. One possible explanation is that the CT98 region may physically interact with its upstream part of FgSnt1 and phosphorylation at S443 releases this self-inhibitory binding and enables its interaction with the Hdf1 HDAC. To test this hypothesis, we generated prey constructs of FgSnt1^N447^ (residues 1–447), FgSnt1^SANT^ (SANT domain, residues 448–1502), and FgSnt1^M633^ (residues 1503–2136) and transformed them in pairs with the bait construct of FgSnt1^CT98^ in to yeast strain AH109. The resulting yeast transformants expressing the FgSnt1^CT98^ bait and FgSnt1^N447^, FgSnt1^SANT^, or FgSnt1^M633^ prey constructs failed to grow on SD-Trp-Leu-His plates and lacked LacZ activities (Figs 11A and S10).

To determine the effect of S443 phosphorylation, we then introduced the S443D and S443A mutations into the prey construct of *FgSNT1*^N447^. The resulting FgSnt1^S443D^ and FgSnt1^S443A^ constructs were co-transformed with FgSnt1^CT98^ into yeast strain AH109. Yeast transformants expressing the FgSnt1^CT98^ bait and FgSnt1^S443D^ prey constructs grew on SD-Trp-Leu-His plates and had LacZ activities. However, yeast transformants expressing the FgSnt1^CT98^ bait and FgSnt1^S443A^ prey constructs failed to grow on SD-Trp-Leu-His plates (Fig 11A), indicating the importance of S443D mutation on the interaction between FgSnt1^N447^ and FgSnt1^CT98^. To confirm this observation, we generated the FgSnt1^N447^-HIS, and FgSnt1^S443D^-HIS and FgSnt1^CT98^-GST fusion proteins. In GST pull down assays, interactions also were only observed between FgSnt1^S443D^-HIS and FgSnt1^CT98^-GST (Fig 11B). These results suggest that the C-terminal tail region of FgSnt1 specifically interacts with its N-terminal region after phosphorylation of S443 by PKA, which may affect the Set3 complex and Hdf1 HDAC activities.

### C-terminal region deletion and S443D mutation of *FgSNT1* rescues the genes expression in the *pkr* mutant though H4 acetylation

**Fig 12:**
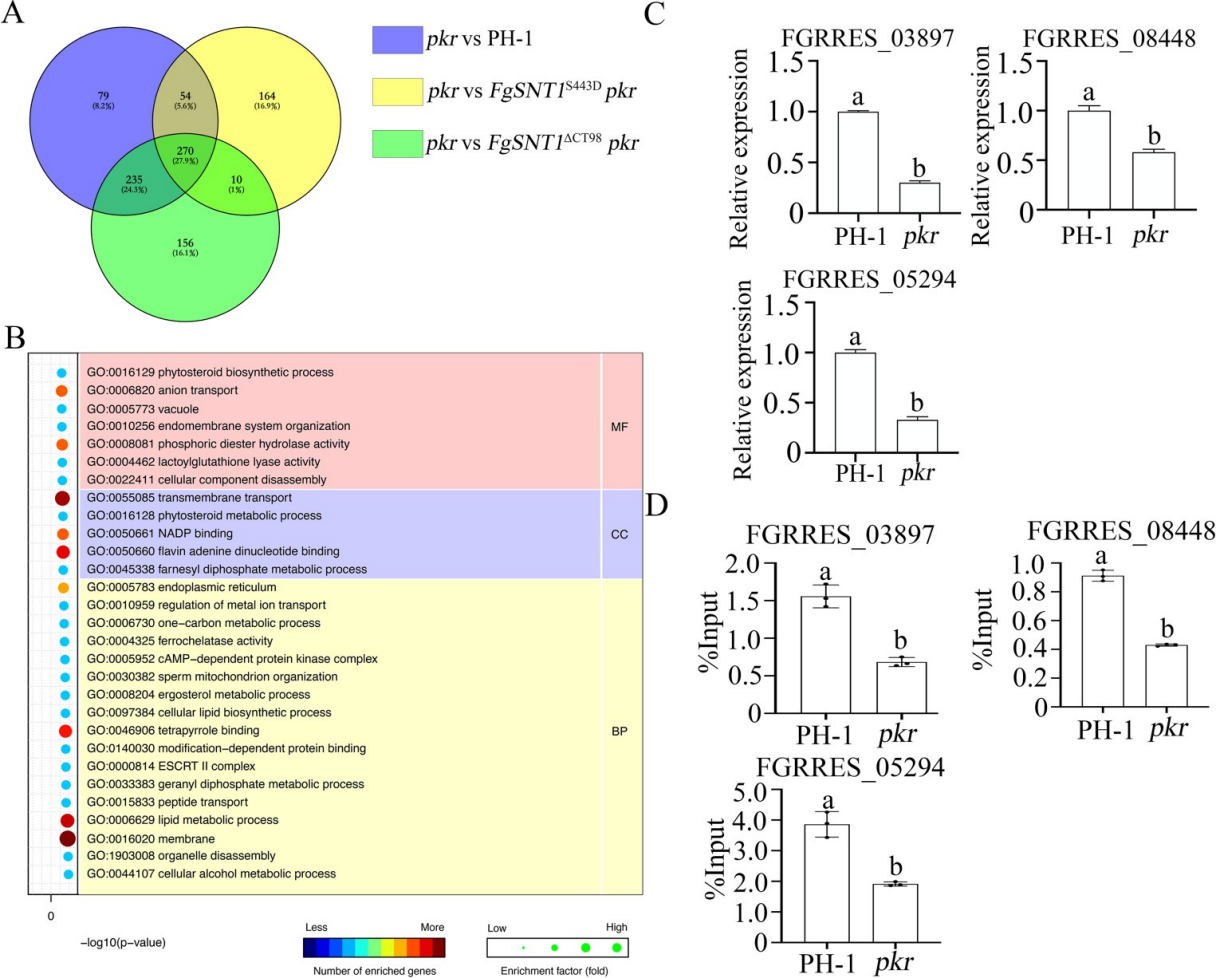
RNA-seq and ChIP-seq analyses with the *pkr1* mutant and its transformants expressing *FgSNT1*^ΔCT98^ and *FgSNT1*^S443D^. **(A)**. A Venn diagram showing the numbers of genes down-regulated (left panel) in the *pkr*, *FgSNT1*^ΔCT98^ *pkr*, and *FgSNT1*^S443D^ *pkr* mutants. **(B)**. GO enrichment analysis of genes down-regulated in the *pkr* mutant but recovered in expression in the *FgSNT1*^ΔCT98^ *pkr* and *FgSNT1*^S443D^ *pkr* mutants. **(C).** The expression levels of FGRRES_08448, FGRRES_03897, and FGRRES_05294 in PH-1 (arbitrarily set to 1) and the *pkr* mutant were assayed by qRT-PCR. **(D)**. The abundance of the promoters of marked genes in the input samples (no ChIP) and chromatin fragments immunoprecipitated with the anti-H4ac antibody in ChIP assays with PH-1 and the *pkr* mutant was assayed by qPCR. Relative abundance (y-axis) is expressed as the percentage of abundance in input samples in immunoprecipitated samples. Different letters (a, b) indicate significant differences based on ANOVA analysis followed by Duncan’s multiple range test (P = 0.05).

Because histone acetylation is associated with gene expression, we used the RNA-seq approach to identify genes affected by deletion of *PKR* and mutation in *FgSNT1*. RNA samples were isolated from hyphae of the wild type, *pkr*, *FgSNT1*^S443D^ *pkr*, and *FgSNT1*^ΔCT98^ *pkr* mutants harvested from YEPD cultures at 12 h. In comparison with the wild type, 638 differentially expressed genes (DEGs) were down-regulated over 2-fold in the *pkr* mutant (S4 Table). Among these DEGs, 270 (27.9%) had their expression increased to the wild-type levels in both *FgSNT1*^S443D^ *pkr*, and *FgSNT1*^ΔCT98^ *pkr* mutants (S4 Table, Fig 12A). GO enrichment analysis showed that those 270 genes were enriched for genes involved in integral component of membrane, carbohydrate transport, metabolic process, and cellular component disassembly (Fig 12B) and 204 of them are in the H3K27me3-enriched chromosomal regions [8]. Interestingly, *CPK1* and the *PTH11* homolog (FGRRES_03897) are among these DEGs. In *M*. *oryzae*, *PTH11* encodes a G-protein coupled receptor (GPCR) that functions upstream the cAMP-PKA pathway for regulating appressorium formation [42] FGRRES_03897, FGRRES_05294, and FGRRES_08448 were selected for verification by qRT-PCR assays and confirmed to be down-regulated in the *pkr* mutant and up-regulated in *FgSNT1*^S443D^ *pkr*, and *FgSNT1*^ΔCT98^ *pkr* transformants (Fig 12C). FGRRES_05294 is predicted to encode an arrestin, which is involved in regulation signal transduction related to GPCRs [43] and FGRRES_08448 encodes an arrestin-domain containing protein.

To determine the regulatory role of H4 acetylation on the expression of FGRRES_08448, FGRRES_03897, and FGRRES_05294, we isolated chromatin fragments of PH-1 and the *pkr* mutant for chromatin precipitation (ChIP) with an anti-H4ac antibody as described [44,45]. Genomic DNAs extracted from chromatin fragments isolated by ChIP or without ChIP (input control) were then used for qPCR assays with primers amplifying the promoter sequences of these three target genes. In comparison with the input control, ChIP with the anti-H4ac antibody enriched the promoter of FGRRES_08448 by 0.91% in PH-1 but only 0.43% in the *pkr* mutant (Fig 12D). Similarly, the level of enrichment for the promoters of FGRRES_03897 and FGRRES_05294 by ChIP with the anti-H4ac antibody was lower in the *pkr* mutant than in PH-1 (Fig 12D). These results suggested that changes in the expression level of FGRRES_08448, FGRRES_03897, and FGRRES_05294 are associated with H4 acetylation, which is affected by deletion of PKR in *F*. *graminearum*.

## Discussion

Like in other fungal pathogens, the cAMP-PKA pathway is involved in regulating hyphal growth, conidiation, ascosporogenesis, and plant infection in *F*. *graminearum* and mutants deleted of its key components have severe defects in growth, pathogenesis, and reproduction. [22,23]. An earlier study showed that 11 of the 67 spontaneous suppressors of *pkr* had mutations in *CPK1* [23]. In this study, we found that Cpk1 proteins were not detectable in the *pkr* mutant. However, treatments with MG132 inhibited Cpk1 degradation. Furthermore, suppressor mutations in *FgBLM10* were identified in 11 suppressor strains, and all of them were nonsense or frameshift mutations. Missense suppressor mutations in *FgPRE5* and *FgPRE6* also were identified in two suppressor strains. In yeast, *PRE5*, *PRE6*, and *BLM10* encode important components of the 26S proteasome. Therefore, it is likely that binding with Pkr regulatory subunits to form inactive holoenzymes is important for protecting Cpk1 proteins from degradation by 26S proteasome in the wild type under normal growth conditions. In the *pkr* mutant, Cpk1 proteins are degraded by 26S proteasome, which may explain similar phenotypes between the *pkr* and *cpk1 cpk2* mutants [22,23]. Suppressor mutations in *FgPRE5*, *FgPRE6*, and *FgBLM10* may negatively impact the activity of 26S proteasome, preventing the degradation of Cpk1 proteins in the absence of Pkr.

Like the *pkr* mutant, the *cpk1 cpk2* mutant also often produces spontaneous suppressors with fast growth rate [26,27]. However, unlike 11 suppressors of *pkr* with mutations in *CPK1*, none of the 30 suppressors of *cpk1 cpk2* sequenced have mutations in *PKR*. Instead, 29 of them have mutations in the transcription factor gene orthologous to Sfl1, which is one of the downstream transcription factors of the cAMP-PKA pathway in *S*. *cerevisiae* [29]. In *M*. *oryzae*, the association of MoSfl1 with the Cyc8-Tup1 transcriptional co-repressor is relieved by phosphorylation in the wild type but disrupted by suppressor mutations in *MoSFL1* the *cpk1 cpk2* mutant [26]. Interestingly, one of the suppressor strains sequenced in this study had a G88D mutation in *FgCYC8* (Table 1), which may impact the expression of genes important for hyphal growth regulated by the Cyc8-Tup1 co-repressor. In addition, we identified one suppressor of *pkr* with a missense mutation in FGRRES_09715, an ortholog of yeast Mig1 (Table 1). In yeast, Mig1 is another one of the downstream transcription factors of PKA that is functionally related to the Cyc8-Tup1 co-repressor for glucose repression [46]. Therefore, suppressor mutations in the direct targets of the cAMP-PKA pathway or Cyc8-Tub1 transcriptional co-repressor may also rescue the growth defect of the *pkr* mutant in *F*. *graminearum*.

Among the 11 *pkr* suppressors with truncation mutations in *FgSNT1*, eight of them had the deletion of C^6595^GCTACC^6601^ and the other three had an insertion of T after C^6065^. The independent recovery of multiple suppressor strains with the same mutations indicates that these are likely hot spots for deletion and insertion mutations. Unfortunately, the flanking DNA sequences of the C^6595^GCTACC^6601^ and C6065 sites have no distinct sequence features that may cause the deletion or insertion except that both of them are in GC-rich regions. Frameshift mutations in these suppressors result in the truncation of C-terminal region of FgSnt1 but do not affect its SANT domains. In this study, we verified that the truncation of C-terminal 98 residues (CT98) caused by the insertion of 7 bp is suppressive to the *pkr* mutant. For the insertion of T that results in the truncation of C-terminal 275 residues (CT275), we did not experimentally verify its suppressive effects on *pkr* because CT98 is a part of CT275.

Snt1 is a key component of the Set3 HDAC complex that is conserved from yeast to human [10,47]. In *F*. *graminearum*, truncation of the C-terminal region of FgSnt1 rescued the defect of *pkr* mutant in H4 acetylation. Furthermore, the Hdf1 HDAC interacted with *FgSNT1*^ΔCT98^ but not with FgSnt1 in yeast two-hybrid and GST pull down assays, indicating that FgSnt1 is also a component of the Set3 complex in *F*. *graminearum*. However, the *snt1* deletion mutant has similar defects with mutants deleted of orthologs of yeast *SIF2* and *HOS2* in *M*. *oryzae*. In *F*. *graminearum*, deletion of *FgSNT1* had no significant effect on vegetative growth and only a minor effect on virulence, which is different from the mutants deleted of the *SIF2* and *HOS2* ortholog [9,12]. Therefore, the *SNT1* orthologs appear to vary in their importance in growth, differentiation, and pathogenesis between *M*. *oryzae* and *F*. *graminearum*, which may be also true to other filamentous fungi or fungal pathogens. Unfortunately, there are only limited studies with *SNT1* orthologs although they are well conserved in fungi. In the fission yeast *Schizosaccharomyces pombe*, Snt1 plays a role in promoting the successful completion of cytokinesis [48]. In the human pathogen *Candida albicans*, Snt1 is important for biofilm formation [33].

Whereas yeast Snt1 is shorter and lacks this tail region, FgSnt1 orthologs from Sordariomycetes, *N*. *crassa* and *M*. *oryzae*, and other *Fusarium* spp. were conserved in the C-terminal 98 amino acids. We showed that the C-terminal 98 aa of FgSnt1 (FgSnt1^CT98^), although not essential for its function, interacted with its N-terminal region (N-terminal 447 aa, FgSnt1^N447^). This C-terminal tail region lacks any known protein motifs but contains intrinsically disordered regions (IDRs) based on analysis with the PONDR (Predictor of Natural Disordered Regions) program [49]. The C-terminal region of FgSnt1 is proline-rich and glycine-rich (Fig 1B). In comparison with FgSnt1, its orthologs in *N*. *crassa* and *M*. *oryzae* have a longer C-terminal region due to the insertion of multiple glutamine residues. The glutamine-rich region may affect the folding of intrinsically disordered proteins [50] but its role in FgSnt1 is unknown.

The cAMP-PKA signal transduction pathway and Set3 HDAC complex both are well conserved in eukaryotic organisms. Although the underlying mechanisms are not clear, cross-interactions between PKA and Set3 complex have been reported in fungal pathogens. In *Ustilago maydis*, Hos2 act as a downstream component of the cAMP-PKA pathway and a transcriptional regulator of the mating-type genes required for the dimorphic switch and pathogenesis [51]. The Set3 complex functions as a key negative regulator of PKA signaling for morphogenesis and virulence in *C*. *albicans* [52]. In this study, we found that Cpk1 interacted with the N-terminal 744 aa of FgSnt1 in *F*. *graminearum*, which shows the physical interaction between a catalytic subunit of PKA with a key component of the Set3 HDAC complex. Furthermore, S443, a conserved PKA phosphorylation site in this region was found be phosphorylated by Cpk1, showing the phosphorylation of a Set3 complex component by PKA. To our knowledge, phosphorylation of Snt1 by PKA and their direct interaction have not been reported in other organisms. It is likely that the physical interaction and functional relationship between PKA and FgSnt1 are conserved in other filamentous fungi.

In *F*. *graminearum*, expression of the phosphorylation-mimic *FgSNT1*^S443D^ allele, like truncation of the C-terminal region of FgSnt1, were suppressive to the *pkr* mutant. In yeast two-hybrid assays and GST pull down assays, FgSnt1^CT98^ only interacted with FgSnt1^N447^ with the S443D mutation. The S443D mutation in *FgSNT1* also affected its interaction with Hdf1. Therefore, PKA may play a direct role in regulating the interaction between the N-terminal and C-terminal regions of FgSnt1 by phosphorylation at S443. The phosphorylation of S443 by PKA may result in the self-inhibitory binding of FgSnt1 via its two terminal regions, which may affect its interaction with Hdf1, Set3 HDAC activities, and H4 acetylation. Interestingly, Set3 was identified as potential PKA phosphorylation targets in *C*. *albicans* by phosphoproteomic and bioinformatic analyses [53]. It is possible that FgSet3 and other components of the FgSet3 complex may also be phosphorylated by PKA in *F*. *graminearum*.

Although the S443D mutation in *FgSNT1* was suppressive to the *pkr* mutant, the *FgSNT1*^S443D^ *pkr* transformant grew slower and produced fewer conidia in comparison with the *FgSNT1*^ΔCT98^ *pkr* mutant, suggesting that the S443D mutation has a weaker suppressive effect than truncation of CT98. One possible explanation is that FgSnt1 may have other phosphorylation sites that may be phosphorylated by PKA [54]. Because Cpk1 also interacted with FgSnt1^745-1490^, additional PKA phosphorylate site, for example S1325 which also fit the PKA phosphorylation motif, may be present between residues 745 and 1490. In yeast, treatments with nocodazole induce the phosphorylation of Snt1 by CDK1, which affects its conformational shifts and roles in establishing activated chromatin states [51]. FgSnt1 may be also subjected to phosphorylation by other protein kinases in regulating genes specific for different developmental and infection stages in *F*. *graminearum*.

A total of 638 genes were downregulated over two-fold in the *pkr* mutant. For 79% and 35% of these genes, their expression was increased to the wild-type level in the *FgSNT1*^ΔCT98^ *pkr* and *FgSNT1*^S443D^ *pkr* transformants, respectively. The number of genes with recovered expression levels by the ΔCT98 and S443D mutations in *FgSNT1* in the *pkr* mutant background are consistent with the degree of recovery in growth rate in these two transformants. Nevertheless, the expression of 79 DEGs downregulated in the *pkr* mutant was not rescued by the ΔCT98 and S443D mutations in *FgSNT1*, suggesting that Pkr and FgSnt1 play distinct roles in regulating their expression. Furthermore, the level of H4 acetylation known to be correlated with gene expression [55,56] was significantly reduced in the *pkr* mutant but recovered in the *FgSNT1*^ΔCT98^ *pkr* transformant. For all three genes selected for ChIP-qPCR analysis, the degree of enrichment of their promoters by ChIP with an anti-H4ac was significantly reduced in the *pkr* mutant compared to PH-1. These results indicate that a subset of genes regulated by Pkr are controlled via FgSnt1 and H4 acetylation in *F*. *graminearum*.

Overall, results from this study suggest that PKA regulates Hdf1 or all Set3 HDAC activities via phosphorylating FgSnt1. In the wild type, unphosphorylated FgSnt1 has no direct interaction with Hdf1. Phosphorylation of S443 by PKA results in the interaction of FgSnt1 between its N-terminal and C-terminal regions and possibly conformation all changes, which leads to the interaction of FgSnt1-S443^P^ with Hdf1 (similar to FgSnt1^ΔCT98^). The interaction of FgSnt1 with Hdf1 may reduce HDAC activities of Hdf1 or FgSet3 complex. Proper regulation of the H4 acetylation level is important for hyphal growth and other developmental or infection processes. In the *pkr* mutant, the absence of Pkr regulatory subunits causes the degradation of Cpk1 by 26S proteasome. Therefore, the *pkr* and *cpk1* mutants had similar defects. When Hdf1 or FgSet3 HDAC activities are not properly regulated by PKA via FgSnt1 in these mutants, a significant reduction in H4 acetylation and reduced expression of genes regulated by H4 acetylation result in growth defects in *F*. *graminearum*. Based on this hypothesis, mutations in other FgSet3 complex components may also be suppressive to the *pkr* mutant. Therefore, it will be important to sequence other suppressor strains for mutations in other key components of the FgSet3 complex, including *FgSET3*, *FTL1* (*SIF2* ortholog), and *HDF1*.

## Materials and methods

### Strains and culture conditions

The wild-type strain PH-1 and all the mutants of *F*. *graminearum* generated in this study were routinely cultured on potato dextrose agar (PDA) at 25°C and assayed for growth rate as described [57]. Conidiation and conidium morphology were assayed with conidia harvested from 5-day-old liquid carboxymethyl cellulose (CMC) cultures [58]. To induce self-fertilization, aerial hyphae of 7-d-old carrot agar (CA) cultures were pressed down with sterile 0.1% Tween 20 and incubated at 25°C. Perithecium formation were examined at seven days after self-fertilization as described [59]. For transformation of *F*. *graminearum*, protoplast preparation and PEG-mediated transformation were performed as described [60]. For transformant selection, hygromycin B and geneticin (Coolaber, Beijing, China) were added to the final concentration of 300 and 200 μg ml^−1^ to both top and bottom agar for selection.

### Identification of suppressor mutations

To identify mutations in *pkr* suppressor strains, genomic DNA was isolated from hyphae harvested from 24 h YEPD cultures and sequenced by Illumina HiSeq 2500 with the Illumina platform at Novogene (Beijing, China) to 50×coverage with pair-end libraries. The resulting genomic sequence data were deposited in the NCBI SRA repository (bioproject accession No. PRJNA855364). The sequence reads were mapped onto the reference genome of strain PH-1 [31]. The variants in all the genome were firstly found by the DNAseq pipeline in hits package (https://github.com/xulab-nwafu/hits), which integrated bowtie2, Picard, SAMtools, and BCFtools [61,62]. The specific mutations in the suppressor genomes were then identified by vcfcmp (https://github.com/xulab-nwafu/filter_vcf), which wrapped the intersection function of BCFtools and variant annotation function of SnpEff [63]. To identify mutations in *FgSNT1* in the other suppressors of *pkr*, its coding region was amplified and sequenced.

### Generation of the *Fgsnt1* and *FgSNT1*^ΔCT98^ deletion mutants

To generate *Fgsnt1* deletion mutant with the split marker approach, the flanking sequences of *FgSNT1* were amplified from PH-1 and connected to fragments of the neomycin resistance marker amplified from pFL2 [64] by overlapping PCR. The resulting *FgSNT1* gene replacement construct was transformed into protoplasts of PH-1. Geneticin-resistant transformants were screened by PCR for the deletion of *FgSNT1*. The same approach was used to generate the *FgSNT1*^ΔCT98^ mutant in which only the C-terminal 98 aa residues of *FgSNT1* (instead of the entire gene) were deleted.

To generate the *Fgsnt1 pkr* and *FgSNT1*^ΔCT98^ *pkr* double mutants, the flanking sequences of *PKR* were amplified and connected to the hygromycin phosphotransferase (*hph*) cassette [22] and transformed into the *Fgsnt1* and *FgSNT1*^ΔCT98^ mutants. Transformants resistant to both hygromycin and geneticin were screened for the deletion of *PKR* and verified for the deletion of *FgSNT1* or its CT98 region. All the primers used to generate and identify these mutants were listed in S2 Table.

### Generation the *FgSNT1*^S443D^ allele and transformants

The S443D mutation in *FgSNT1* was introduced by overlapping PCR as described [26] with primers list in the S2 Table. The resulting PCR products were cloned into vector pKNTG carrying the geneticin-resistance marker [65]. The *FgSNT1*^S443D^ construct was then transformed into protoplasts of *Fgsnt1* mutant that was generated by replacing the ORF of *FgSNT1* with the hygromycin phosphotransferase (*hph*) and thymidine kinase cassettes (Human Simplex Virus, HSV-*tk*). Transformants resistant to geneticin and floxuridine (5-Fluorouracil 2’-deoxyriboside) were screened by PCR to identify *FgSNT1*^S443D^ transformants and verified by sequencing analysis for the S443D mutation. The *PKR* gene replacement construct carrying *hph* was transformed into protoplasts of the *FgSNT1*^S443D^ mutant to generate the *FgSNT1*^S443D^ *pkr* mutants.

### Plant infection assays

Flowering wheat heads of 6-week-old wheat cultivar XiaoYan 22 were inoculated with 10 μl of conidia suspensions (2×10^4^ conidia ml^−1^) at the fifth spikelet from the base as described [57]. Spikelets with typical wheat scab symptoms were examined at 14 dpi to estimate the disease index [24]. For assaying infectious growth, infected rachis tissues were embedded in Spurr resin after fixation and dehydration as described previously [23]. Thick sections were then prepared and stained with 0.5% (wt/vol) toluidine blue as described [66]. For assaying infection cushion formation, infected lemmas were fixed with 4% (vol/vol) glutaraldehyde and dehydrated in a series of acetone. The samples were coated with gold–palladium and examined with a JEOL 6360 scanning electron microscope (Jeol Ltd., Tokyo, Japan) as described [66].

### Detection of histone acetylation levels

Total proteins were isolated from vegetative hyphae harvested from 12 h YEPD cultures as described [24]. Acetylation of histone H3 and H4 was detected with anti-H3ac (ab47915), anti-H4ac (ab177790), and anti-H4K16ac (ab194352) antibodies from Abcam (Cambridge, UK). Detection with the anti-H3 (ab209023, Abcam), and anti-H4 (ab10158, Abcam) antibodies were used as the loading controls. The band intensity was quantified using ImageJ software (National Institutes of Health, Bethesda, MD, USA). Relative intensity of the histone acetylation was normalized to that of non-acetylated histone, and compared with that of the control group [67]. Each experiment was performed for three independent biological replicates, and error bars represent standard deviation estimated with data from three independent replicates.

### Assays for PKA activity and Cpk1 expression

Vegetative hyphae were harvested from 24 h YEPD (Yeast extract peptone dextrose) cultures by filtration through two layers of Miracloth (Sigma, USA) and washed with sterile water [25]. PKA activities were assayed with the PKA kinase assay kits (Type I) (IMMUNECHEM, China) [68]. The total protein isolated from *F*. *graminearum* and ATP solution were added to the 96 wells plate, which was pre-immobilized with 50 μL of PKA-substrate (KRREILSRRPSYR). After the kinase reaction, the PKA-substrate in wells were mixed with anti-pSubstrate antibodies and then incubated with anti-rabbit IgG HRP solution with TMB (3,3’-5,5’-Tetramethylbenzidine). The PKA phosphorylation activity is proportional to the color intensity. The primary antibody to *F*. *graminearum* Cpk1 was generated in rabbits using the synthetic oligopeptide VKAGAGDASQFDRYPE (ABclonal, Wuhan, China). The specificity of the resulting anti-Cpk1 antibody was verified by western blot analysis with total proteins isolated from the *cpk1* mutant [23] and PH-1 as described [36,69]. Detection with an anti-Tub2 β-tubulin antibody [70] was used as the loading control.

### Yeast two-hybrid assays

The Matchmaker yeast two-hybrid system (Clontech, Mountain View, CA, USA) was used to assay protein-protein interactions. The *FgSNT1* and *FgSNT1*^ΔCT98^ ORFs were amplified from the 1^st^-strand cDNA synthesized with the HiScript II Q RT SuperMix (Vazyme Biotech, Nanjing, China) as described [71] and cloned into pGBK7 as the bait vector. The prey construct of *HDF1* and *FgSET3* were generated with pGADT7 (Clontech). The resulting bait and prey vectors were co-transformed in pairs into yeast strain AH109 (Clontech). The Leu^+^ Trp^+^ transformants were isolated and assayed for growth on SD-Trp-Leu-His medium and galactosidase activities with filter lift assays [72]. The positive and negative controls were provided in the Matchmaker library construction kit (Clontech).

### GST pull-down assays

To generate the *FgSNT1*-GST and *FgSNT1*^ΔCT98^-GST fusion constructs, their ORFs were amplified from 1^st^ strand cDNA synthesized with RNA isolated from PH-1 and *FgSNT1*^ΔCT98^ mutant as described [71] and cloned into pGEX4T1 [73]. To generate *HDF1*-HIS constructs, its ORFs were amplified from PH-1 by RT-PCR and cloned into pCOLDI [74]. Recombinant Hdf1-HIS, FgSnt1-GST, and FgSnt1^ΔCT98^-GST fusion proteins were isolated from *Escherichia coli* cells as described with anti-GST beads [75]. Equal amounts of Hdf1-HIS and FgSnt1-GST or FgSnt1^ΔCT98^-GST fusion proteins were mixed and incubated at 4°C for 4 h before adding glutathione resins (Smart Lifesciences, Changzhou, China). After incubation for 12 h and washing for three times, proteins bound to glutathione resins were eluted as described [76,77]. Western blots of total proteins and proteins eluted from glutathione resin were detected with the anti-GST (#CW0084) and anti-HIS (#CW0286) antibodies from CWBIO (Beijing, China). The *FgSNT1*^1-774^-GST and Cpk1-HIS fusion constructs were generated with the same approach. Equal amounts of Cpk1-HIS and *FgSNT1*^1-774^-GST fusion proteins were mixed and incubated with glutathione resins (Smart Lifesciences, Changzhou, China) [76,77]. Western blots of total proteins and proteins eluted from glutathione resin were detected with the anti-GST (#CW0084) and anti-HIS (#CW0286) antibodies from CWBIO (Beijing, China).

### Phos-tag gel assays

The *FgSNT1*^1-774^-GST, and Cpk1-HIS fusion constructs were generated from the cDNA of PH-1 as described [71] and cloned into pGEX4T1 and pCOLDI [73,74]. Recombinant *FgSNT1*^1-774^-GST and Cpk1-HIS fusion proteins were isolated and incubated with 75 μM ATP solution (TakaRa) for 2 h as described [38]. Samples were boiled for 5 min prior to loading onto a 10% SDS-PAGE gel and Phos-tag SDS-PAGE, respectively. SDS-PAGE was carried out with polyacrylamide gels. Phos-tag SDS-PAGE was performed with 7.5% polyacrylamide gels containing 20 μM Phos-tag acrylamide and 40 μM MnCl_2_ [39]. After electrophoresis, proteins were transferred to PVDF membranes (Sigma) and probed with an anti-GST antibody. The phosphorylation status of *FgSNT1*^1-774^-GST was analyzed based on the changes in the mobility shifts of phosphorylated proteins on Phos-tag SDS-PAGE. To determine the critical phosphorylated sites, FgSnt1^S18A^-, FgSnt1^S38A^-, FgSnt1^S322A^-, FgSnt1^S443A^-, and FgSnt1^S511A^-GST were constructed by overlapping PCR with primers list in the S2 Table. Similar approaches were used to determine the phosphorylation of these potential phosphorylated residues.

### Quantitative interaction studies by isothermal titration calorimetry (ITC)

Protein-protein interactions were analyzed according to the enthalpy changes produced in titration of Cpk1-HIS binding with FgSnt1^1-744^-GST with the isothermal titration calorimetry instrument (NanoITC, New Castle, USA). Specifically, 50 μL of 0.1 mM Cpk1 was loaded into an ITC syringe and titrated into an ITC sample cell containing 300 μL of 0.01 mM FgSnt1^1-744^-GST dissolved solution at a sequence of 25 injections. The control experiments including the titration of Cpk1-HIS to PBS, titration of PBS to PBS and titration of PBS to FgSnt1^1-744^-GST were performed, in which the enthalpy change produced in the titration of Cpk1 to PBS was subtracted from that produced in the titration of Cpk1-HIS to the FgSnt1^1-744^-GST (S7 Fig) [78].

### RNA-seq analysis

Vegetative hyphae of PH-1 and the *pkr* mutant, *FgSNT1*^ΔCT98^ *pkr*, and *FgSnt1*^S443D^ *pkr* mutants were harvested from 12 h liquid YEPD cultures. Total RNA was extracted with the TRIzol Reagent (Life technologies, US) following the manufacturer’s instructions. Library construction and sequencing with an Illumina Nova-PE150 sequencer were performed at Novogene (Beijing, China). Over 25 Mb high-quality RNA-seq reads (deposited at the NCBI Sequence Read Archive database under the bioproject accession No. PRJNA858617) were obtained for each sample and mapped onto the reference genome of *F*. *graminearum* [31] with HISAT2[79]. The number of reads mapped to each gene was analyzed with the featureCounts software [80]. DEGs were identified using the edgeRun package [81] with the log2 FC (log_2_ fold change) greater than 1 and false discovery rate (FDR) less than 0.05 as the cut off values and analyzed with Blast2GO for GO enrichment [82] with the *p*-values calculated by the Benjamini-Hochberg procedure [83]. All the Perl, R, and Shell scripts used in this study for sequencing and other analysis were available at GitHub as described [7].

### ChIP-qPCR assays

Vegetative hyphae of PH-1 and the *pkr* mutant were harvested from 12 h liquid YEPD cultures. Chromatins were cross-linked with 0.75% formaldehyde for 15 min at room temperature [84]. Samples were ground with liquid nitrogen and re-suspended in lysis buffer (50 mM, HEPES-KOH pH 7.5, 140 mM NaCl, 1 mM EDTA PH 8.0, 1% Triton, 0.1% Sodium Deoxycholate, 1% SDS) with protease inhibitors (Sigma, USA). The cross-linked chromatins were sonicated by Vibra-Cell (Sonics, USA) to an average DNA fragment size of 100–500 bp. An aliquot of the sheared lysate was saved as the input control for each sample. The rest was incubated with 10 μg anti H4ac antibody (ab177790, Abcam) and protein A agarose beads (Cell Signaling Technology, USA) as described [44]. Genomic DNA in the immunocomplexes formed on agarose beads was recovered with the HiPure Gel Pure DNA Mini Kit (Megan, China). The presence of the promoter sequences of targeted genes in the input and recovered ChIP samples was assayed by quantitative PCR with the ChamQ SYBR qPCR Master Mix (Vazyme, China) with primers listed in S2 Table. The resulting ChIP data were analyzed with the Percent Input Method [85].

## Supporting information

S1 Table Mutations identified by sequencing PCR products in the *pkr* suppressor strains. (DOC)

S2 Table Primers used in this study. (DOCX)

S3 Table Putative PKA Phosphorylation sites in FgSnt1. (DOCX)

S4 Table RNA-seq data analysis of of wild type PH-1, *pkr* mutant, *FgSNT1*^S443D^ *pkr*, and *FgSNT1*^ΔCT98^ *pkr* mutants. (XLSX)

S1 Fig Assay of growth rate of all spontaneous *pkr* suppressors. 60 subcultures of spontaneous sectors were collected and categorized into three types based on their growth rate. 14 type I suppressor strains grew more than two-fold faster than *pkr* mutant. 34 type II suppressors grew 1.5-fold faster than *pkr* mutant. 12 type III suppressors grew less than 1.5-fold faster than *pkr* mutant.
(TIF)

S2 Fig Phenotypes of the spontaneous suppressor strains selected for whole genome sequencing. **(A).** Three-day-old PDA cultures of the wild-type strain PH-1, *pkr* mutant and *pkr* suppressors **(B).** Perithecia from mating cultures of the marked strains were examined at 12 dpf. **(C).** Flowering wheat heads were drop-inoculated with conidia of the marked strains and photographed 14 days post-inoculation (dpi). Black dots mark the inoculated spikelets.
(TIF)

S3 Fig Schematic diagram of the primers used to generate mutants in *Fusarium graminearum*. (A). Schematic diagram of the primers used to generate *Fgsnt1* mutants (left panel), and screening of *Fgsnt1* transformants by PCR amplification (right panel). (B). Schematic diagram of the primers used to generate *FgSNT1*^ΔCT98^ transformants (left panel), and screening of *FgSNT1*^ΔCT98^ transformants by PCR amplification (right panel). (C). Schematic diagram of the primers used to generate *pkr* mutant in *Fgsnt1* and *FgSNT1*^ΔCT98^ mutants (left panel), and screening of *pkr* transformants by PCR amplification (right panel). (D). Schematic diagram of the primers used to introduce S443D mutation. (E). Schematic diagram of the primers used to generate *FgSNT1*^S443D^ gene replacement constructs.
(JPG)

S4 Fig Deletion of the of *FgSNT1* have no H4 acetylation affection in the *pkr* mutant. **(A).** Western blots of total proteins isolated from PH-1 and 5 independent *pkr* mutants were detected with antibodies specific for H4ac. Detection with anti-H4 antibodies was used as a loading control. **(B).** Western blots of total proteins isolated from PH-1, *FgSNT1*^ΔCT98^ *pkr* and *pkr* mutants were detected with antibodies specific for H4ac. Detection with anti-H4 antibodies was used as a loading control**. (C).** Western blots of total proteins isolated from PH-1 and the *pkr*, *Fgsnt1 pkr*, and *Fgsnt1*mutants were detected with antibodies specific for H4ac. Detection with anti-H4 antibodies was used as a loading control.
(TIF)

S5 Fig Assays for PKA activities and Cpk1 expression in suppressor H10. **(A).** Western blots of total proteins isolated from hyphae of PH-1, *pkr*, *cpk1*, and suppressor strain H10 were detected with an anti-Cpk1 antibody. **(B).** PKA activities were assayed with proteins isolated from the marked strains.
(TIF)

S6 Fig Yeast two-hybrid assays for the interaction of FgSnt1 with Pkr and Cpk1. The negative controls with empty prey or bait constructs were presented to support the results of Yeast two-hybrid assays in Fig 7.
(TIF)

S7 Fig The ITC thermogram of binding interactions between Cpk1 and FgSnt1^1-744^. The control experiments were performed including the titration of Cpk1-HIS to PBS (A), titration of PBS to PBS (B), and titration of PBS to FgSnt1^1-744^-GST (C). The enthalpy changes produced in titration of Cpk1-HIS binding with FgSnt1^1-744^-GST were analyzed accordingly (D).
(TIF)

S8 Fig Predicted phosphorylation sites in the FgSnt1. Schematic drawing of the FgSnt1 protein and alignment of its predicted phosphorylation sites with orthologs from *F*. *graminearum* (Fg), *F*. *oxysporum* (Fo), *M*. *oryzae* (Mo), and *N*. *crassa* (Nc). The predicted phosphorylation sites were labeled with stars.
(TIF)

S9 Fig *In vitro* kinase assays for the phosphorylation of FgSnt1^1-744^ by Cpk1. In vitro kinase assays with Cpk1-HIS and FgSnt1^S18A^-GST, FgSnt1^S38A^-GST, FgSnt1^S322A^-GST, or FgSnt1^S511A^-GST fusion proteins. The S18, S38, S322, or S511 to A mutation in FgSnt1^1-744^-GST did not change its phosphorylation by Cpk1.
(TIF)

S10 Fig Yeast two-hybrid Assays for intramolecular interactions in FgSnt1. Yeast two-hybrid assays with cells expressing the empty prey or bait constructs as the negative controls for Fig 11.
(TIF)
