# Supplementary material for: FgSnt1 of the Set3 HDAC complex plays a key role in mediating the regulation of histone acetylation by the cAMP-PKA pathway in Fusarium graminearum
Source: PLoS Genet. 2022 Dec 7;18(12):e1010510. doi: 10.1371/journal.pgen.1010510 (PMC9728937; doi:10.1371/journal.pgen.1010510)
Supplement: S1 Table — (DOC) [file pgen.1010510.s001.doc]

**S1 Table. Mutations identified by sequencing PCR products in the *pkr* suppressor strains**

|  | **Fg00324** | **Fg01210** | **Fg07282** | **Fg05222** | **Fg09715** | **Fg12901** | **Fg05396** | **Fg00385** | **Fg07251** | **Fg04083** | **Fg03942** | **Fg05234** | **Fg02424** | **Fg10588** | **Fg10589** | **Fg00887** |
| --- | --- | --- | --- | --- | --- | --- | --- | --- | --- | --- | --- | --- | --- | --- | --- | --- |
| Sc | *SNT1* | *BLM10* | *PRE6* | *PRE5* | *MIG1* | *CYC8* | *SGF73* | *NHP6A/6B* | *TPK2* | NA | NA | NA | NA | NA | NA | NA |
| Sp | *SNT1* | NA | *PRE6* | *PRE5/* | *SCR1* | *SSN6* | *SGF73* | *NHP6* | *TPK2* | NA | NA | NA | NA | NA | NA | NA |
| Human | *NCOR1* | *PSME4* | *PSMA7* | *PSMA1* | NA | *KDM6A* | *ATXN7* | *HMGB1* | *CPK1* | NA | NA | NA | NA | NA | NA | NA |
| H1 | - | - | - | - | - | - | - | - | Y575fs | ND | ND | ND | ND | ND | ND | ND |
| H2 | - | - | - | - | - | - | - | - | D561N | ND | ND | ND | G446S | ND | ND | ND |
| H3 | - | - | - | - | - | - | - | - | E406D | ND | ND | P25fs | ND | ND | ND | ND |
| H4 | - | - | D82N | - | - | - | - | - | - | ND | ND | ND | ND | ND | ND | ND |
| H5 | A1958fs | Q1000* | - | - | - | - | - | - | - | ND | ND | ND | ND | ND | ND | ND |
| H6 | - | - | - | K62E | - | - | - | - | - | ND | ND | ND | ND | ND | ND | ND |
| H7 | A1958fs | - | - | - | - | - | - | - | - | ND | ND | ND | ND | ND | ND | ND |
| H8 | - | - | - | - | - | - | - | - | - | ND | ND | ND | ND | ND | ND | ND |
| H9 | - | Q314* | - | - | - | - | - | - | - | ND | ND | ND | ND | ND | ND | ND |
| H10 | - | S392fs | - | - | - | - | - | - | - | ND | ND | ND | ND | ND | ND | ND |
| H11 | A1958fs | G1380fs | - | - | - | - | - | - | - | ND | ND | ND | ND | ND | ND | ND |
| H12 | - | - | - | - | - | - | - | - | - | ND | ND | ND | ND | ND | ND | ND |
| H13 | - | S392fs | - | - | - | - | - | - | - | ND | ND | ND | ND | ND | ND | ND |
| H14 | - |  | - | - | - | - | - | - | H531R | ND | ND | ND | ND | ND | ND | ND |
| H15 | - | L1195fs | - | - | - | - | - | - | - | ND | ND | ND | ND | ND | ND | ND |
| H16 | Y2135fs | - | - |  | - | - | - | - | - | ND | ND | ND | ND | ND | ND | ND |
| H17 | Y2135fs | - | - | - | - | - | - | - | - | ND | ND | ND | ND | ND | ND | ND |
| H18 | - | - | - | - | - | - | - | - | - | ND | ND | ND | ND | ND | ND | ND |
| H19 | - | - | - | - | - | - | - | - | - | ND | ND | ND | ND | ND | ND | ND |
| H20 |  | - | - | - | - | - | - | - | - | ND | ND | ND | ND | ND | ND | ND |
| H21 | - | L1195fs | - | - | - | - | - | - | - | ND | ND | ND | ND | ND | ND | ND |
| H22 | - | - | - |  | - | G88D | - | - | - | ND | ND | ND | ND | del | del | del |
| H23 | - | F396fs | - | - | - | - | - | - | - | ND | ND | ND | ND | ND | ND | ND |
| H24 | - | - | - | - | - | G88D | - | - | - | ND | ND | ND | ND | ND | ND | ND |
| H25 | - | L317fs | - | - | - | - | - | - | - | ND | ND | ND | ND | ND | ND | ND |
| H26 | Y2135fs | - | - | - | - | - | - | - | - | ND | ND | ND | ND | ND | ND | ND |
| H27 | - | - | - | - | - | - | - | - | - | ND | ND | ND | ND | ND | ND | ND |
| H28 | - | - | - | - | - | - | - | - | - | ND | R140M | ND | ND | ND | ND | ND |
| H29 | Y2135fs | - | - | - | - | - | - | - | - | ND | ND | ND | ND | ND | ND | ND |
| H30 | - | - | - | - | - | - | - | L57P | - | M368fs | ND | ND | ND | ND | ND | ND |
| H31 | - | - | - | - | - | - | - | - | - | ND | ND | ND | ND | ND | ND | ND |
| H32 | - | L1195fs | - | - | - | - | - | - | - | ND | ND | ND | ND | ND | ND | ND |
| H33 | - | - | - | - | - | - | - | - | - | ND | ND | ND | ND | ND | ND | ND |
| H34 | - | - | - | - | - | - | - | - | delete L^310^ to V^315^ | ND | ND | ND | ND | ND | ND | ND |
| H35 | - | - | - | - | - | - | - | - | - | ND | ND | ND | ND | ND | ND | ND |
| H36 | - | - | - | - | - | - | - | - | - | ND | ND | ND | ND | ND | ND | ND |
| H37 | - | - | - | - | - | - | - | - | - | ND | ND | ND | ND | ND | ND | ND |
| H38 | - | - | - | - | - | - | - | - | - | ND | ND | ND | ND | ND | ND | ND |
| H39 | - | - | - | - | - | - | - | - | - | ND | ND | ND | ND | ND | ND | ND |
| H33 | - | - | - | - | - | - | - | - | - | ND | ND | ND | ND | ND | ND | ND |
| H34 | - | - | - | - | - | - | - | - | - | ND | ND | ND | ND | ND | ND | ND |
| H35 | - | - | - | - | - | - | R257K | - | - | ND | ND | ND | ND | ND | ND | ND |
| H36 | - | - | - | - | - | - | - | - | - | ND | ND | ND | ND | ND | ND | ND |
| H37 | - | - | - | - | - | - | - | - | - | ND | ND | ND | ND | ND | ND | ND |
| H38 | - | - | - | - | - | - | - | - | - | ND | ND | ND | ND | ND | ND | ND |
| H39 | - | - | - | - | - | - | - | - | - | ND | ND | ND | ND | ND | ND | ND |
| H40 | - | - | - | - | - | - | - | - | - | ND | ND | ND | ND | ND | ND | ND |
| H41 | - | - | - | - | - | - | - | - | - | ND | ND | ND | ND | ND | ND | ND |
| H42 | - | - | - | - | - | - | - | - | - | ND | ND | ND | ND | ND | ND | ND |
| H43 | - | - | - | - | - | - | - | - | - | ND | ND | ND | ND | ND | ND | ND |
| H44 | Y2135fs | - | - | - | - | - | - | - | - | ND | ND | ND | ND | ND | ND | ND |
| H45 | - | - | - | - | - | - | - | - | - | ND | ND | ND | ND | ND | ND | ND |
| H46 | - | - | - | - | - | - | - | - | - | ND | ND | ND | ND | ND | ND | ND |
| H47 | - | N799fs | - | - | - | - | - | - | - | ND | ND | ND | ND | ND | ND | ND |
| H48 | - | - | - | - | - | - | - | - | - | ND | ND | ND | ND | ND | ND | ND |
| H49 | - | - | - | - | - | - | - | - | - | ND | ND | ND | ND | ND | ND | ND |
| H50 | - | - | - | - | - | - | - | - | - | ND | ND | ND | ND | ND | ND | ND |
| H51 | Y2135fs | - | - | - | - | - | - | - | - | ND | ND | ND | ND | ND | ND | ND |
| H52 | Y2135fs | - | - | - | - | - | - | - | - | ND | ND | ND | ND | ND | ND | ND |
| H53 | Y2135fs | - | - | - | - | - | - | - | - | ND | ND | ND | ND | ND | ND | ND |
| H54 | - | - | - | - | - | - | - | - | E590* | ND | ND | ND | ND | ND | ND | ND |
| H55 | - | - | - | - | - | - | - | - | K529N | ND | ND | ND | ND | ND | ND | ND |
| H56 | - | - | - | - | - | - | - | - | - | ND | ND | ND | ND | ND | ND | ND |
| H57 | - | - | - | - | K71E | - | - | - | - | ND | ND | ND | ND | ND | ND | ND |
| H58 | - | - | - | - | - | - | - | - | - | ND | ND | ND | ND | ND | ND | ND |
| H59 | - | - | - | - | L291fs | - | - | - | - | ND | ND | ND | ND | ND | ND | ND |
| H60 | - | - | - | - | - | - | - | - | - | ND | ND | ND | ND | ND | ND | ND |

-, sequenced but no changes found

*, stop codon

fs, frameshift mutation

del, delete the whole reading frame of gene

ND, not detected
