# Supplementary material for: FgSnt1 of the Set3 HDAC complex plays a key role in mediating the regulation of histone acetylation by the cAMP-PKA pathway in Fusarium graminearum
Source: PLoS Genet. 2022 Dec 7;18(12):e1010510. doi: 10.1371/journal.pgen.1010510 (PMC9728937; doi:10.1371/journal.pgen.1010510)
Supplement: S2 Table — (DOCX) [file pgen.1010510.s002.docx]

**S2 Table. Primers used in this study**

| Name | Sequence(5’-3’) |
| --- | --- |
| BLM10-seq-F | ATGGACGAGAACATCGGACCT |
| BLM10-seq-R | GGCAAAGTAACTCTTCCAGAGTACTCC |
| SNT1-seq-F | ATGGAGTCCCAGTCGCTTAC |
| SNT1-seq-R | TCATCTTCTACTATAAGGATCCGGATGT |
| PRE5-seq-F | ATGTTTCGAAACAACTACGATAACGATTC |
| PRE5-seq-R | TTAGCTGTCAACGTCCATACCTTC |
| PRE6-seq-F | ATGGTGGATCCCAGCTCCG |
| PRE6-seq-R | TTATTCCGCAGCAGAGTCATCTTGA |
| MIG1-seq-F | ATGCAACGAGCACAGTCGG |
| MIG1-seq-R | TTACATGCGATCCATGAGATCGCC |
| CYC8-seq-F | ATGGCGAACCATCACGCG |
| CYC8-seq-R | TCATTCAACTTTTGGGGCGGC |
| SGF73-seq-F | ATGGGGTCAGACGCTCGAAAAGG |
| SGF73-seq-R | CTATGCTCTGCTTGAAACCGAGGG |
| NHP6-seq-F | ATGCCTAAGGCTGCTGCTC |
| NHP6-seq-R | CTAAGAAGACTCCTCTTCTTCCTGATCG |
| SNT1 H2R | TTGACCTCCACTAGCTCCAGCCAAGCCGCGAGACGCTAGACCTTGAAT |
| SNT1 H3F | GAATAGAGTAGATGCCGACCGCGGGTTGCAAGCAAACATGGCGATAGT |
| SNT1 H4R | TCGGTTCAGTTTGTTGATGTTGG |
| SNT1 H5F | GCCCAAAGCTCCGAAATCAAAC |
| SNT1 H6R | CTCCTTCCTCGCCCTCCTGT |
| SNT1 H7F | CCAATCCTCAAAGGCACAGTAGT |
| SNT1 H8R | ATGCCAACCTCCTCCAAGTG |
| SNT1C-1FNEW | CCAGGAATGCCACCTCTC |
| SNT1C-2RNEW | CAGATACGGCAGAGAAATCGCAACCTCACCCTGCATCTGATGTTGC |
| SNT1C-3FNEW | GTTTAGATTCCAAGTGTCTACTGCTGGCGCAAGCAAACATGGCGATAGT |
| SNT1C-3FNEW | TCGGTTCAGTTTGTTGATGTTGG |
| SNT1C-4RNEW | GCCTCCAGTCTCGCG |
| SNT1C-5FNEW | GAGTTCGAGAGACATCCGG |
| SNT1C-6RNEW | TCCACTCCCAGGACCAGC |
| SNT1C-7FNEW | ATGCCAACCTCCTCCAAGTG |
| SNT1C-8RNEW | TTCCTCCCTTTATTTCAGATTCAA |
| H855/R | GCTGATCTGACCAGTTGC |
| H856/F | GTCGATGCGACGCAATCGT |
| H852 | ATGTTGGCGACCTCGTATTGG |
| H850 | TTCCTCCCTTTATTTCAGATTCAA |
| HYG/f | GGCTTGGCTGGAGCTAGTGGAGGTCAA |
| HY/r | GTATTGACCGATTCCTTGCGGTCCGAA |
| HT-F | ACAGAAGATGATATTGAAGGAGC |
| HT-R | GTCGACTTAATAACACATTGCGGACGT |
| G852/F | TCGGCTATGACTGGGCACAACA |
| G850/R | GAGCGGCGATACCGTAAAGCAC |
| G855/R | TGTTGGGTTTGAGCTAGGTGGG |
| G856/F | GAATGGTCAAATCAAACTGCTAGATAT |
| GEN/F | GAGGTTGCGATTTCTCTGCCGTATCTG |
| GEN/R | GCCAGCAGTAGACACTTGGAATCTAAAC |
| GE/R | CAGTCGATGAATCCAGAAAAGCG |
| EN/F | GGAAGGGACTGGCTGCTATTGG |
| PKR1F | TGTGCGTCAACTATGGGTCTGG |
| PKR2R newHPH | AATGCTCCTTCAATATCATCTTCTGTCAGATGTTATGTCGTCGAAGAGGC |
| PKR 3F newHPH | CGTCCGCAATGTGTTATTAAGTCGACAGTGATGTTCAGGTTGTCTCGTG |
| PKR 4R | AGCCAGGAGAAATAACGCACC |
| PKR 5F | ACGACACCATCACATCGCCTAC |
| PKR 6R | AGATCCCTTCTCGACGACATAGAA |
| PKR 7F | GCCTTGTGAGCCCTTGTTGTG |
| PKR 8R | GGAAACCGAAGTTGCTGTTGAGG |
| SNT1-BD-F | GAGGACCTGCATATGATGGCTCAAAGATACCCACGTTAC |
| SNT1-BD-R | CATAACAGCAGTTTTAGAGCCC |
| SNT1-BD-2F | GGGCTCTAAAACTGCTGTTATGGTGAAAAATTATTATGTCCGTC |
| SNT1-full-R | CTCCATGGCCATATGTCATCTTCTACTATAAGGATC |
| SNT1-c98-R | CTCCATGGCCATATGGTAGCGACCCTGCATCTGAT |
| Hdf1-AD-F  Hdf1-AD-R  Set3-AD-F  Set3-AD-R | ATCCATCGAGCTCGAATGGATATAGACTCCTACAGGTATCGG  CATCTGCAGCTCGAGGAGTTGCATTGCAGTTCCTACC  ATCCATCGAGCTCGAATGACCGAAAAACCGGCG  CATCTGCAGCTCGAGTAAGGAGCCATTTGTCGCCG |
| SNT1^S443D^-F | ATCTTCAAACTCGAGATGTAAGGTTACAACATCATCCCACT |
| SNT1^S443D^-R | TACTGAGAATCACCACGTCGC |
| SNT1^S443D^-2F | ACGTGGTGATTCTCAGTACGA |
| SNT1^S443D^-2R | ACCGTCGACCTCGAGTCTTCTACTATAAGGATCCGGATGTCTCT |
| SNT1^S443A^-F | GACGTGGTGCTTCTCAGTACGAT |
| SNT1^S443A^-R | TACTGAGAAGCACCACGTCGCATAG |
| AD-N447-F | ATCCATCGAGCTCGAATGGCTCAAAGATACCCACGT |
| AD-N447-R | CATCTGCAGCTCGAGATCGTACTGAGAACTACCACGT |
| AD-N443D-R | CATCTGCAGCTCGAGATCGTACTGAGAATCACCACGT |
| BD-C98-F | GAGGACCTGCATATGATGCGCTACCCGCCT |
| BD-C98-R | CTCCATGGCCATATGTCATCTTCTACTATAAGGATCCGGATGT |
| AD448-1502-F | GCTATGCGACGTGGTGATTCTCAGTACGATCATTATAACGAGGATGAGAGGCG |
| AD448-1502-R | ACGGACATAATAATTTTTCACCATAACAGCAGTTTTAGAGCCCATG |
| AD1503-2136-F | TCTAAAACTGCTGTTATGGTGAAAAATTATTATGTCCGTCAAAAGGACC |
| AD1503-2136-R | CTCCATGGCCATATGTCATCTTCTACTATAAGGATCCGGATGT |
| Snt1-one-GST-F | ATCTGGTTCCGCGTGGATCCATGGCTCAAAGATACCCACGT |
| Snt1-one-GST-R | TCGAGTCGACCCGGGAATTCAAGCTGTGTCTCGGCTG |
| Snt1-two-GST-F | ATCTGGTTCCGCGTGGATCCATGGAACCCATCGCGCAAC |
| Snt1-two-GST-R | TCGAGTCGACCCGGGAATTCAACAGCAGTTTTAGAGCCCATG |
| Snt1-three-GST-F | ATCTGGTTCCGCGTGGATCCATGGTGAAAAATTATTATGTCCGTCAAAAGG |
| 4T-1-BamHI-SNT1-F | GTTCCGCGTGGATCCATGGCTCAAAGATACCCACGTTAC |
| 4T-1-Xhol-SNT1-R | ATGCGGCCGCTCGAGTTCATCTTCTACTATAAGGATC |
| 4T-1-Xhol-SNT1^C98^-R | GTAGCGACCCTGCATCTGATACTCGAGCGGCCGCAT |
| 4T-1-BamHI-SNT1^443D^-R | ATCGTACTGAGAATCACCACGT |
| PCOLD-Set3-F | GCGCGGCAGCATCGAAGGTAGGCATATGACCGAAAAACCGGCGTC |
| PCOLD-Set3-R | GGGAAGGCCGTCGGTGGCCTGCAGGTAAGGAGCCATTTGTCGCCG |
| PCOLD-Hos2-F | GCGCGGCAGCATCGAAGGTAGGCATATGGATATAGACTCCTACAGGTATCGG |
| PCOLD-Hos2-R | GGGAAGGCCGTCGGTGGCCTGCAGGCTAGAGTTGCATTGCAGTTCCTACC |
| S38A-1R | GGCCGGTCGGCG |
| S38A-2F | GTGACAGACGCCGACCGGCC |
| S322A-1R | GGCCAGTCGACCAGC |
| S322A-2F | GGACCTTCGGCTGGTCGACTGGCCATAGGAG |
| S443A-1R | GGCACCACGTCGCATAG |
| S443A-2F | GACCAGCTATGCGACGTGGTGCCTCTCAGT |
| S511A-1R | GGCATCCCGTCTAGAAGCA |
| S511A-2F | TTAATGCTTCTAGACGGGATGCCTATCGCT |
| S1325A-1F | ATCTGGTTCCGCGTGGATCCATGGACTACTTCGATATGGAAATTGGG |
| S1325A-1R | GGCAATCATATCTGGAATGACGG |
| S1325A-2F | GTCATTCCAGATATGATTGCCACACAGGAAGAAAAG |
| S1325A-2R | TCGAGTCGACCCGGGAATTCACCTCCGCGCCTG |
| TK-snt1-7F | TTGAGTTTGTTGAAGTCCAGCT |
| Tk-snt1-1F | TGACGAGGACAGCCTCTG |
| TK-snt1-2R | TTGACCTCCACTAGCTCCAGCCAAGCCTTTTCAAGGGCTGTGGGAAAC |
| TK-snt1-3F | CGTCCGCAATGTGTTATTAAGTCGAC TGCGACGTGGTAGTTCTCAG |
| TK-snt1-4R | GACGAGTCTCCACGGACAT |
| TK-snt1-5F | GTGGGCCCCGTGG |
| TK-snt1-6R | TCCCCCATCGACCCTCTT |
| TK-snt1-8R | GGGGAGGTTTCTGAAGGAGAAT |
| TK-D- mutation F | ACGTGGTGATTCTCAGTACGAT |
| TK-D-mutation R | ATCGTACTGAGAATCACCACGT |
| TK-A-mutation F | CGTGGTGCTTCTCAGTACGA |
| TK-A-mutation R | TCGTACTGAGAAGCACCACG |
| Cpk1-AD-F | GCCATGGAGGCCAGTGAATTCGCCGGTGTGAAGTGGAT |
| Cpk1-AD-R | CCACTGCTTGGGTGGAATTCATGCCTTCACTGGGGTTTCT |
| Pkr-AD-F | GCCATGGAGGCCAGTGAATTCATGTCCGGCCCTTTCTCC |
| Pkr-AD-R | CCACTGCTTGGGTGGAATTCGCCGGTGTGAAGTGGAT |
| CT98-GST-F | ATCTGGTTCCGCGTGGATCC |
| CT98-GST-R | TCGAGTCGACCCGGGAATTC |
| FgSnt1^N447^-HIS-F | CGGCAGCATCGAAGGTAGGCATATGTAAGGTTACAACATCATCCCACT |
| FgSnt1^N447^-HIS-R | GGGAAGGCCGTCGGTGGCCTGCAGGATCGTACTGAGAACTACCACGT |
| FgSnt1^S443D^-HIS-R | GGGAAGGCCGTCGGTGGCCTGCAGGATCACCACGTCGCATAGCTGGT |
| UP-FGRRES_08448-F | GGAGAACATCTGGCCTAACAA |
| UP-FGRRES_08448-R | CGTAGTCTCTTGGGAAGGTTAAG |
| chip-FGRRES_08448-1F | CAGGGGTGGTATCACTGAAC |
| chip-FGRRES_08448-1R | GCCTGATGCTCGCAT |
| chip-FGRRES_08448-2F | AAAGGAAAGCCCATTGCC |
| chip-FGRRES_08448-2R | AGTTATGGATGAGATGGGATTAGAG |
| UP-FGRRES_05294-F | CCCAGTGCTATCATGCTTTCT |
| UP-FGRRES_05294-R | GGGAATGCTCAGGTTGATGT |
| chip-FGRRES_05294-1F | CTTCTCCAACTGATCTGACC |
| chip-FGRRES_05294-1R | GTGTCCTGACGATCCAGA |
| chip-FGRRES_05294-2F | ACCAAATATATCAAGCGTCATTGT |
| chip-FGRRES_05294-2R | GAAGGCAGAGAATATCCATATCAG |
| UP-FGRRES_03897-F | CGAGTTCCTTCGCTATGTCTAC |
| UP-FGRRES_03897-R | AGTGTCCGTCATGCAGTTTAT |
| chip-FGRRES_03897-1F | CGAACGTCTCGGCTTC |
| chip-FGRRES_03897-1R | AAAACGAGCACTAGTTCCC |
| chip-FGRRES_03897-2F | TCAGTCCCAGTCCAGTG |
| chip-FGRRES_03897-2R | AGAGCCTTGTGGAGATCT |
