# Supplementary material for: FgSnt1 of the Set3 HDAC complex plays a key role in mediating the regulation of histone acetylation by the cAMP-PKA pathway in Fusarium graminearum
Source: PLoS Genet. 2022 Dec 7;18(12):e1010510. doi: 10.1371/journal.pgen.1010510 (PMC9728937; doi:10.1371/journal.pgen.1010510)
Supplement: S3 Table — (DOCX) [file pgen.1010510.s003.docx]

**S3 Table. Putative PKA Phosphorylation sites in FgSnt1**

| Sequence | Site Position | PhosphoRS Binomial Peptide Score |
| --- | --- | --- |
| SRSPRDRSPDRFDRG | S18 | 24.850 |
| DGDRRRPSEVRPGNG | S38 | 24.633 |
| GPSAGRLSIGEGYVS | S322 | 24.785 |
| RPAMRRGSSQYDHYN | S443 | 27.206 |
| INASRRDSYRSGPSP | S511 | 27.116 |
| TPGRRGGSVKVDHPE | S1325 | 24.954 |

Note: Predict phosphorylation sites by GPS5.0 and NetPhos-3.1.
