# Supplementary figures and images for: FgSnt1 of the Set3 HDAC complex plays a key role in mediating the regulation of histone acetylation by the cAMP-PKA pathway in Fusarium graminearum
Source: PLoS Genet. 2022 Dec 7;18(12):e1010510. doi: 10.1371/journal.pgen.1010510 (PMC9728937; doi:10.1371/journal.pgen.1010510)

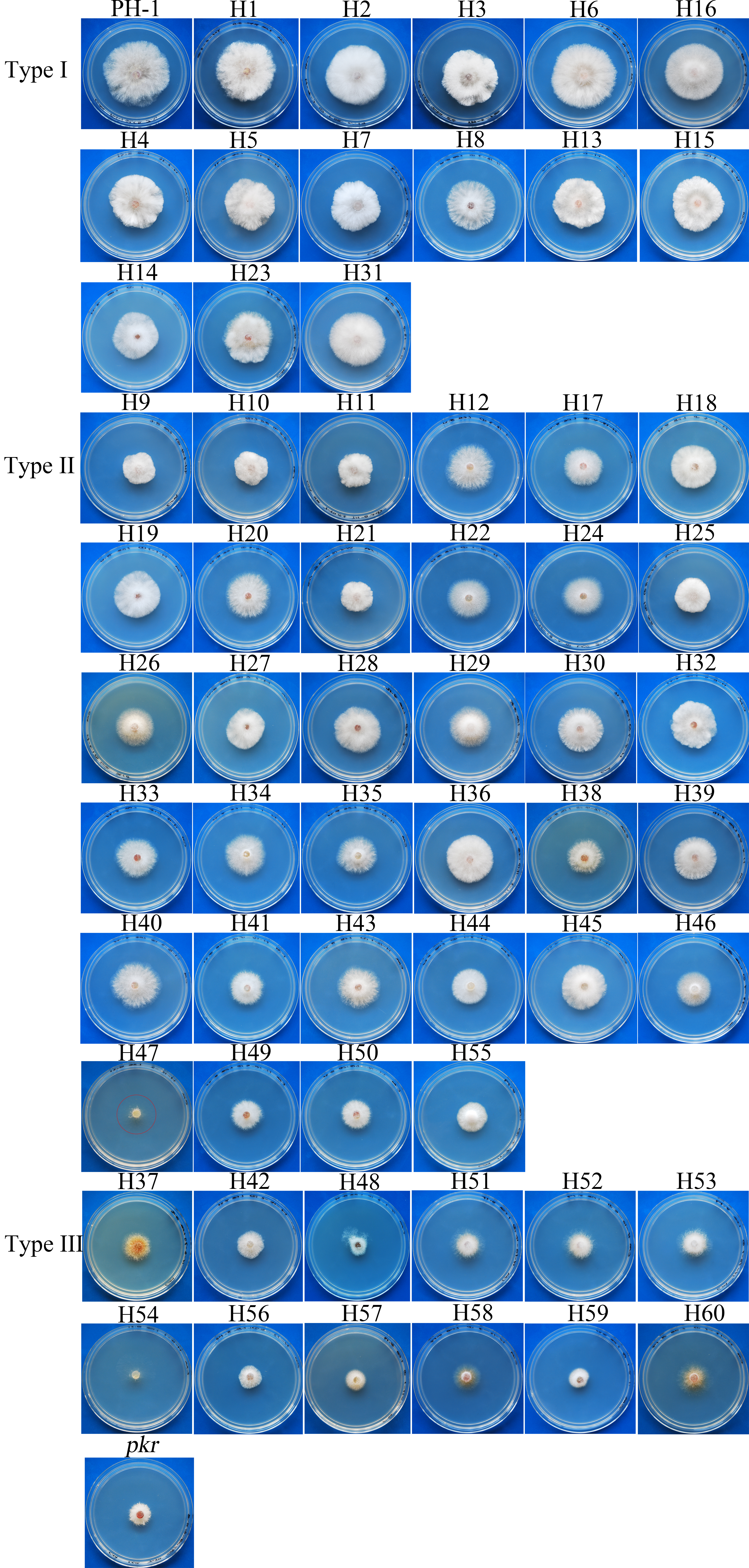

Supplement: S1 Fig — 60 subcultures of spontaneous sectors were collected and categorized into three types based on their growth rate. 14 type I suppressor strains grew more than two-fold faster than pkr mutant. 34 type II suppressors grew 1.5-fold faster than pkr mutant. 12 type III suppressors grew less than 1.5-fold faster than pkr mutant. (TIF) [file pgen.1010510.s005.tif]

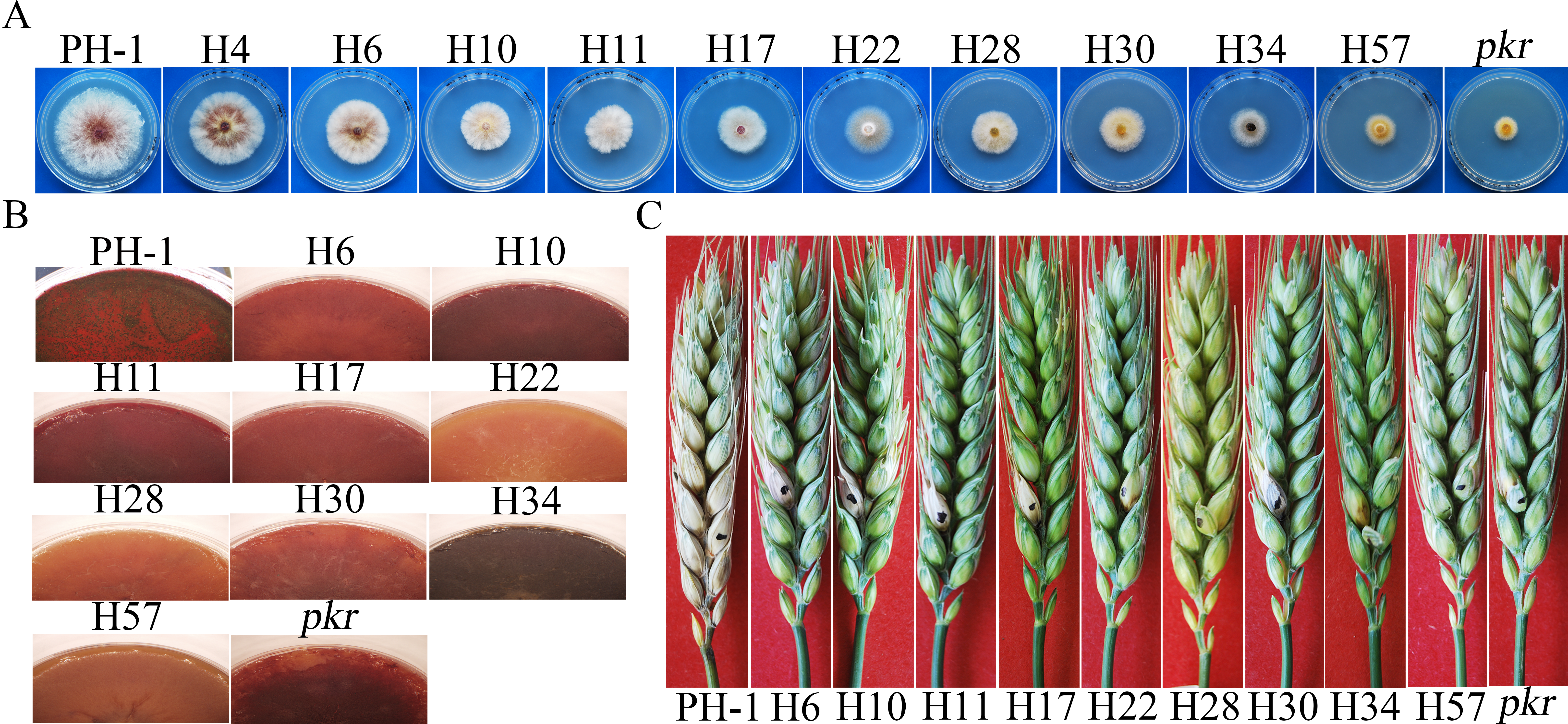

Supplement: S2 Fig — (A). Three-day-old PDA cultures of the wild-type strain PH-1, pkr mutant and pkr suppressors (B). Perithecia from mating cultures of the marked strains were examined at 12 dpf. (C). Flowering wheat heads were drop-inoculated with conidia of the marked strains and photographed 14 days post-inoculation (dpi). Black dots mark the inoculated spikelets. (TIF) [file pgen.1010510.s006.tif]

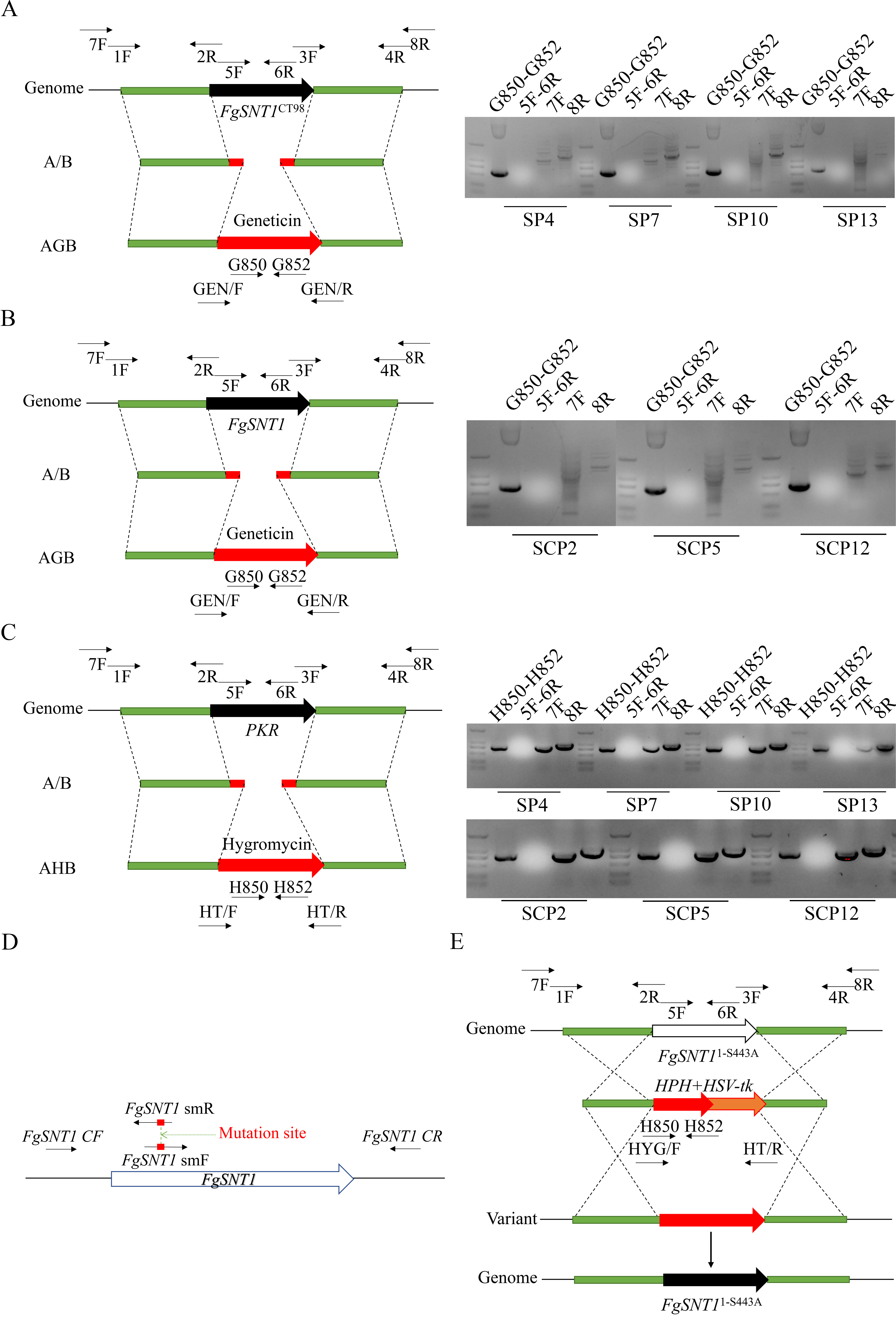

Supplement: S3 Fig — (A). Schematic diagram of the primers used to generate Fgsnt1 mutants (left panel), and screening of Fgsnt1 transformants by PCR amplification (right panel). (B). Schematic diagram of the primers used to generate FgSNT1ΔCT98 transformants (left panel), and screening of FgSNT1ΔCT98 transformants by PCR amplification (right panel). (C). Schematic diagram of the primers used to generate pkr mutant in Fgsnt1 and FgSNT1ΔCT98 mutants (left panel), and screening of pkr transformants by PCR amplification (right panel). (D). Schematic diagram of the primers used to introduce S443D mutation. (E). Schematic diagram of the primers used to generate FgSNT1S443D gene replacement constructs. (JPG) [file pgen.1010510.s007.jpg]

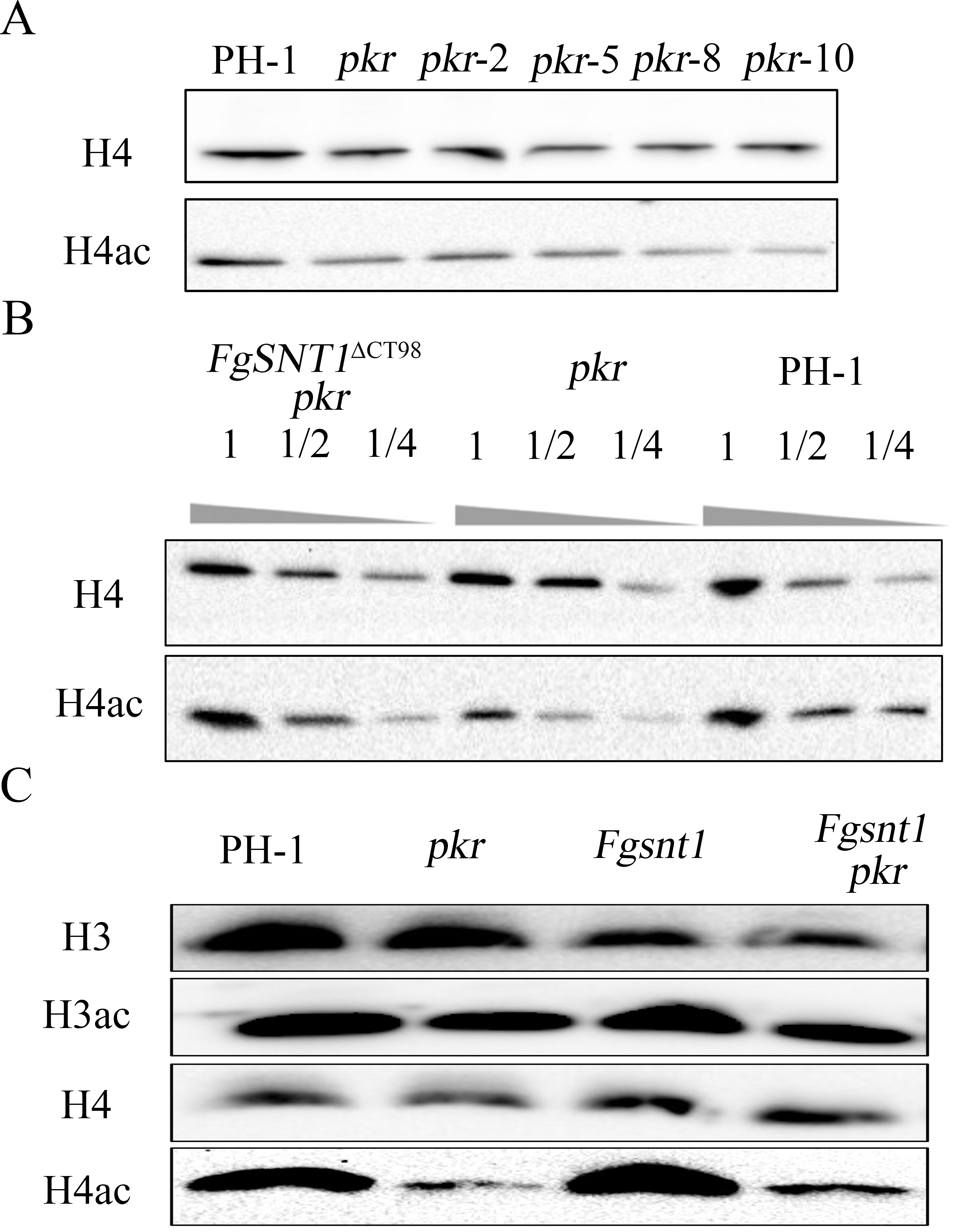

Supplement: S4 Fig — (A). Western blots of total proteins isolated from PH-1 and 5 independent pkr mutants were detected with antibodies specific for H4ac. Detection with anti-H4 antibodies was used as a loading control. (B). Western blots of total proteins isolated from PH-1, FgSNT1ΔCT98 pkr and pkr mutants were detected with antibodies specific for H4ac. Detection with anti-H4 antibodies was used as a loading control. (C). Western blots of total proteins isolated from PH-1 and the pkr, Fgsnt1 pkr, and Fgsnt1mutants were detected with antibodies specific for H4ac. Detection with anti-H4 antibodies was used as a loading control. (TIF) [file pgen.1010510.s008.tif]

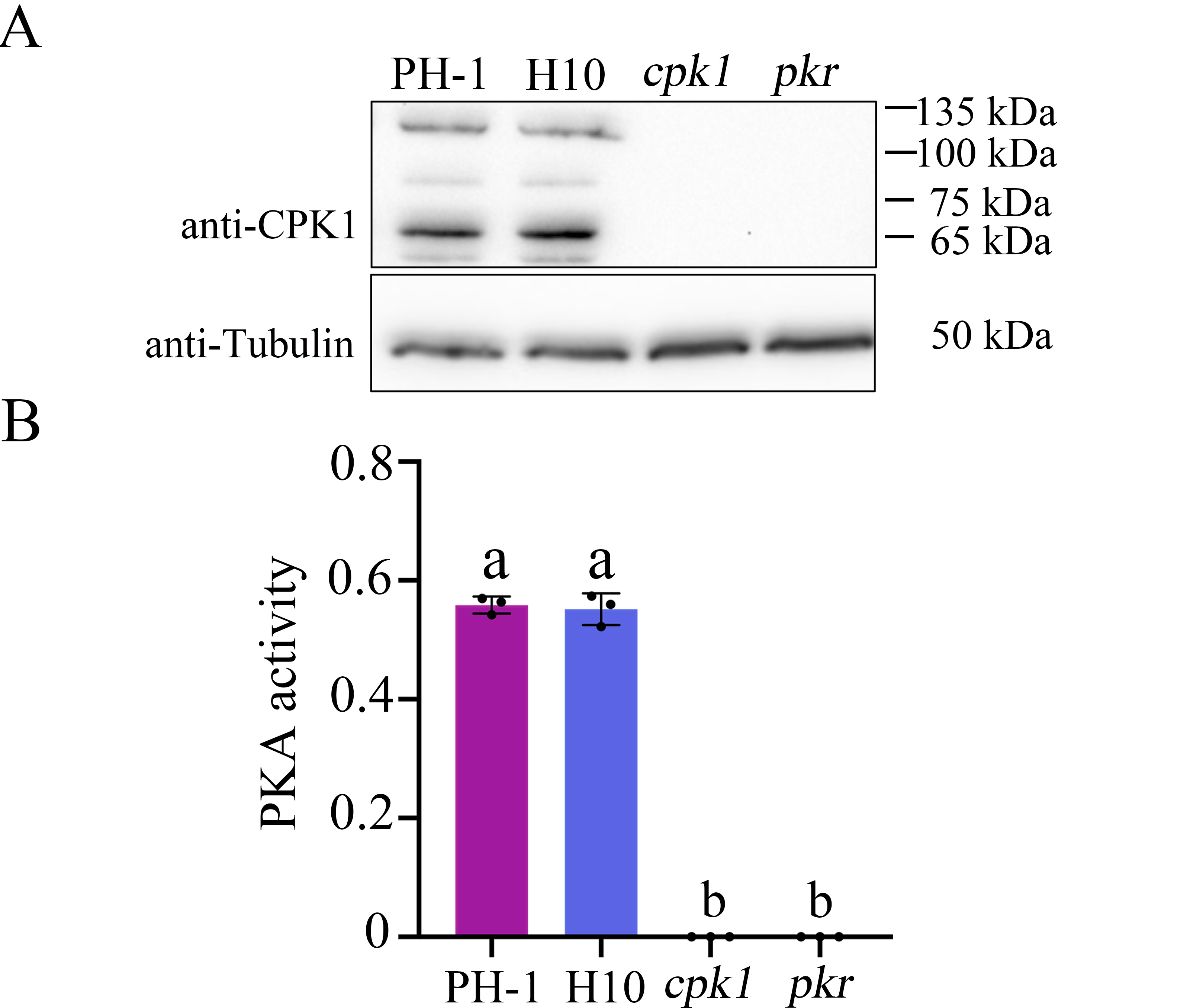

Supplement: S5 Fig — (A). Western blots of total proteins isolated from hyphae of PH-1, pkr, cpk1, and suppressor strain H10 were detected with an anti-Cpk1 antibody. (B). PKA activities were assayed with proteins isolated from the marked strains. (TIF) [file pgen.1010510.s009.tif]

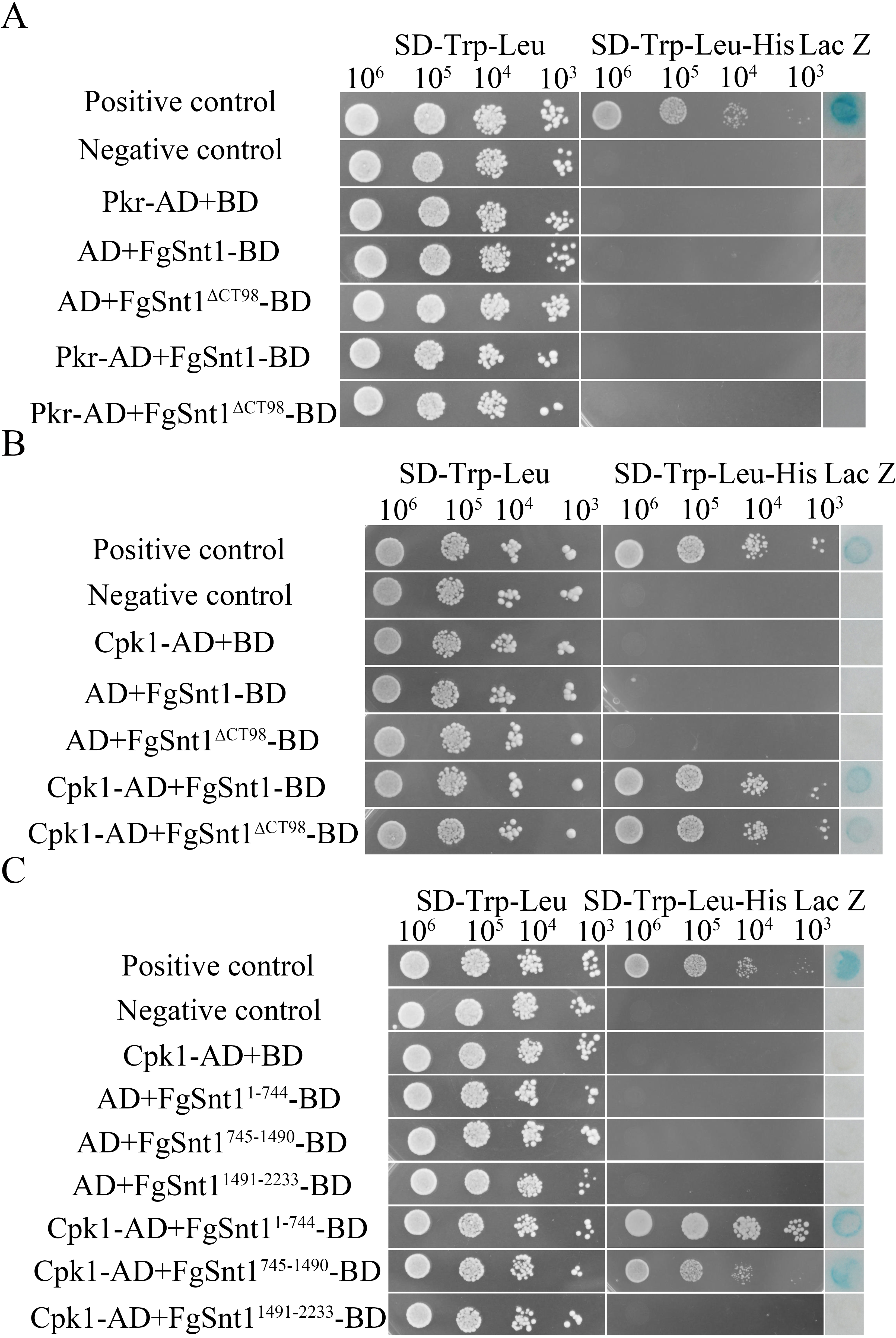

Supplement: S6 Fig — The negative controls with empty prey or bait constructs were presented to support the results of Yeast two-hybrid assays in Fig 7. (TIF) [file pgen.1010510.s010.tif]

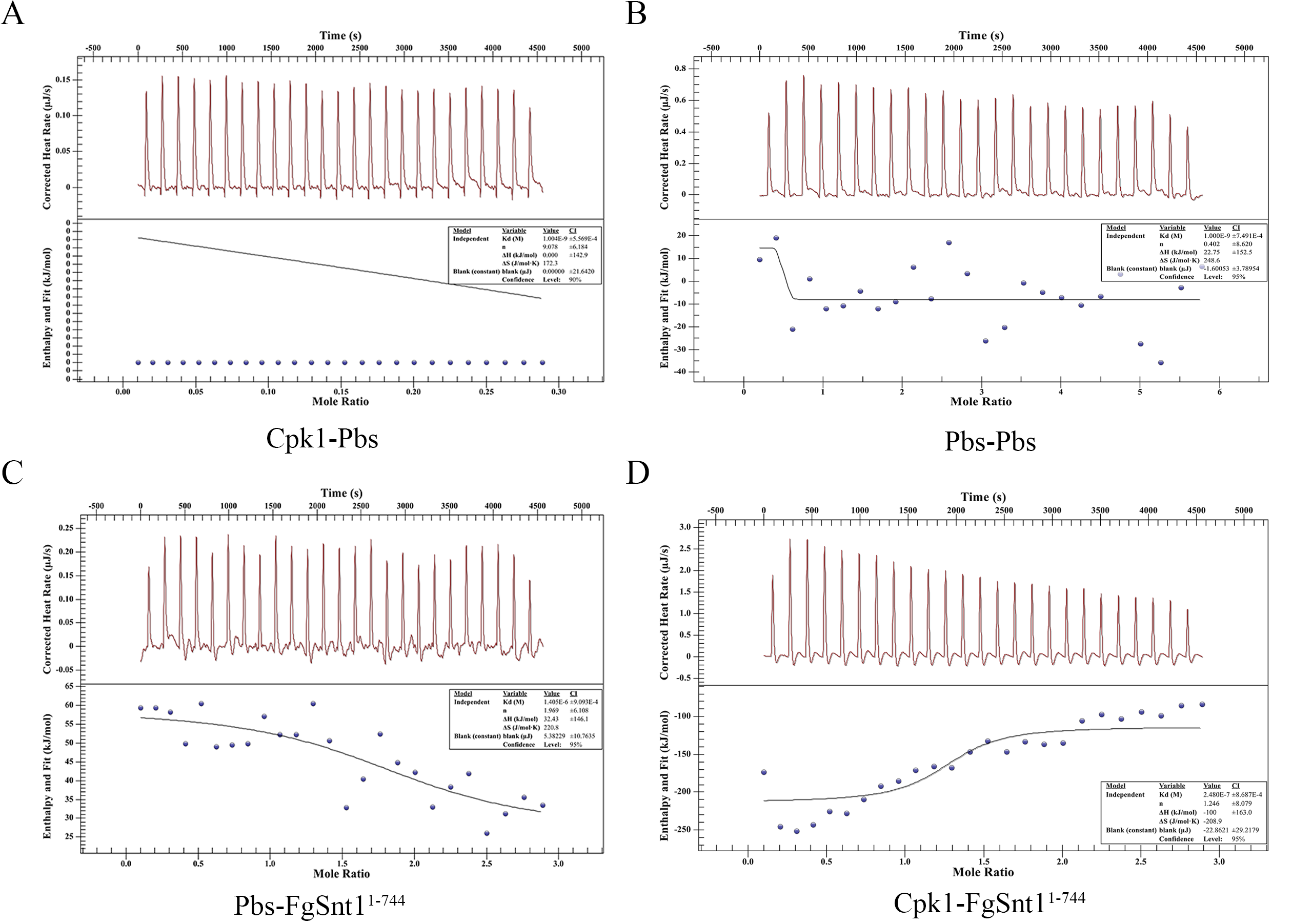

Supplement: S7 Fig — The control experiments were performed including the titration of Cpk1-HIS to PBS (A), titration of PBS to PBS (B), and titration of PBS to FgSnt11-744-GST (C). The enthalpy changes produced in titration of Cpk1-HIS binding with FgSnt11-744-GST were analyzed accordingly (D). (TIF) [file pgen.1010510.s011.tif]

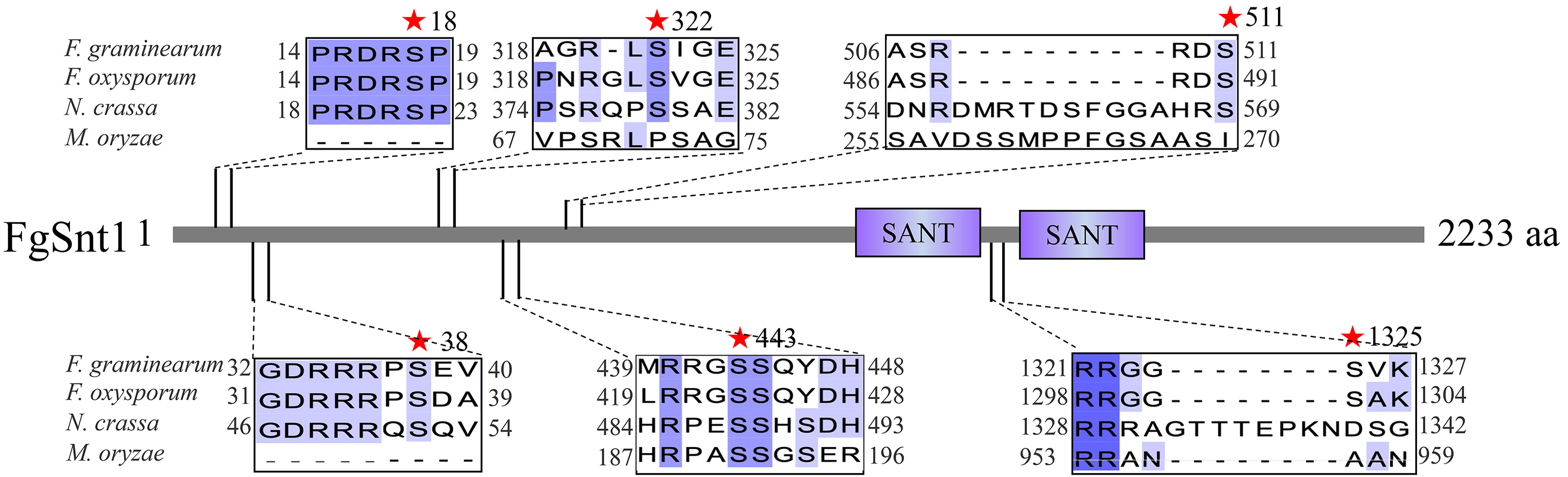

Supplement: S8 Fig — Schematic drawing of the FgSnt1 protein and alignment of its predicted phosphorylation sites with orthologs from F. graminearum (Fg), F. oxysporum (Fo), M. oryzae (Mo), and N. crassa (Nc). The predicted phosphorylation sites were labeled with stars. (TIF) [file pgen.1010510.s012.tif]

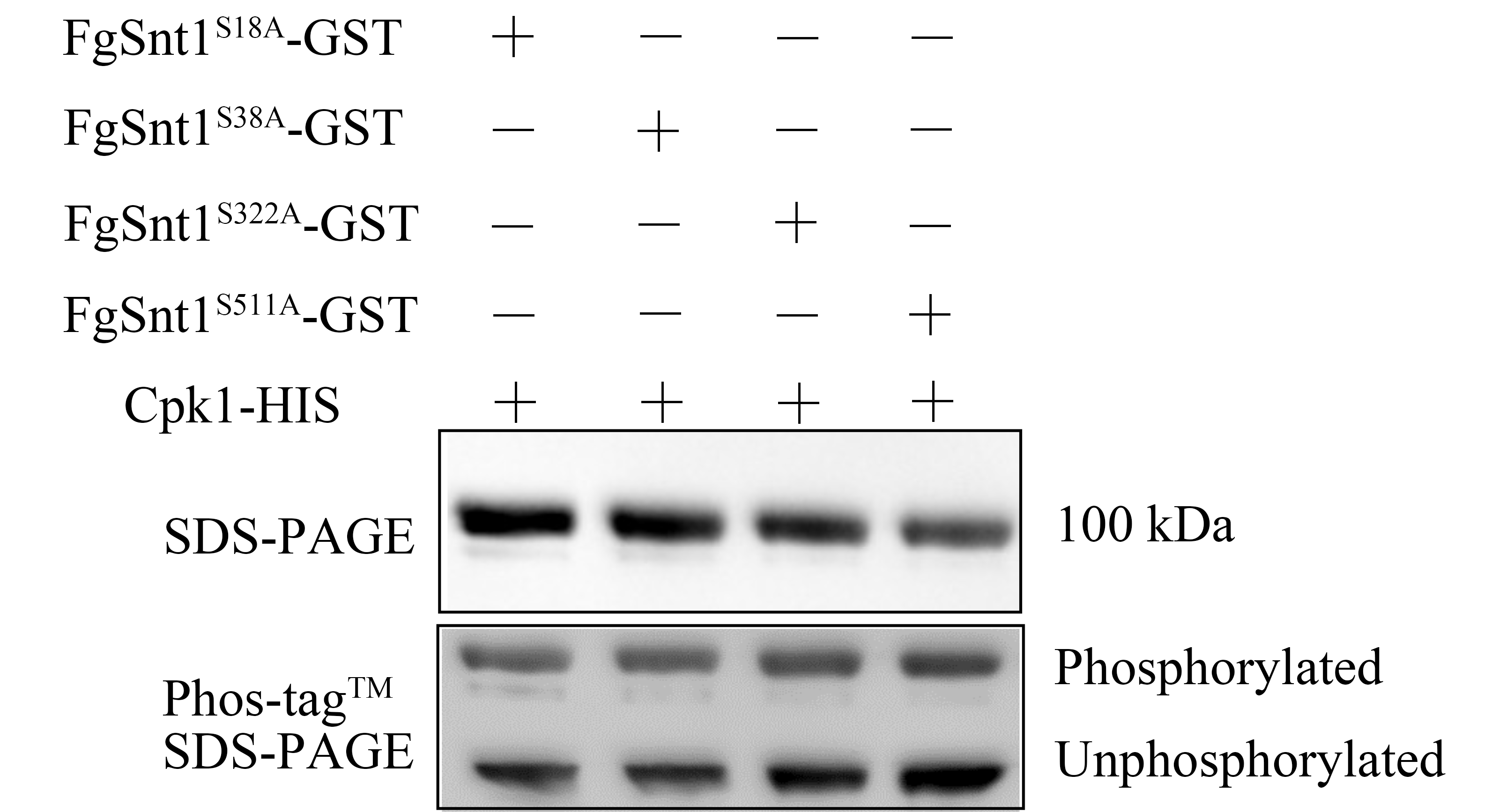

Supplement: S9 Fig — In vitro kinase assays with Cpk1-HIS and FgSnt1S18A-GST, FgSnt1S38A-GST, FgSnt1S322A-GST, or FgSnt1S511A-GST fusion proteins. The S18, S38, S322, or S511 to A mutation in FgSnt11-744-GST did not change its phosphorylation by Cpk1. (TIF) [file pgen.1010510.s013.tif]

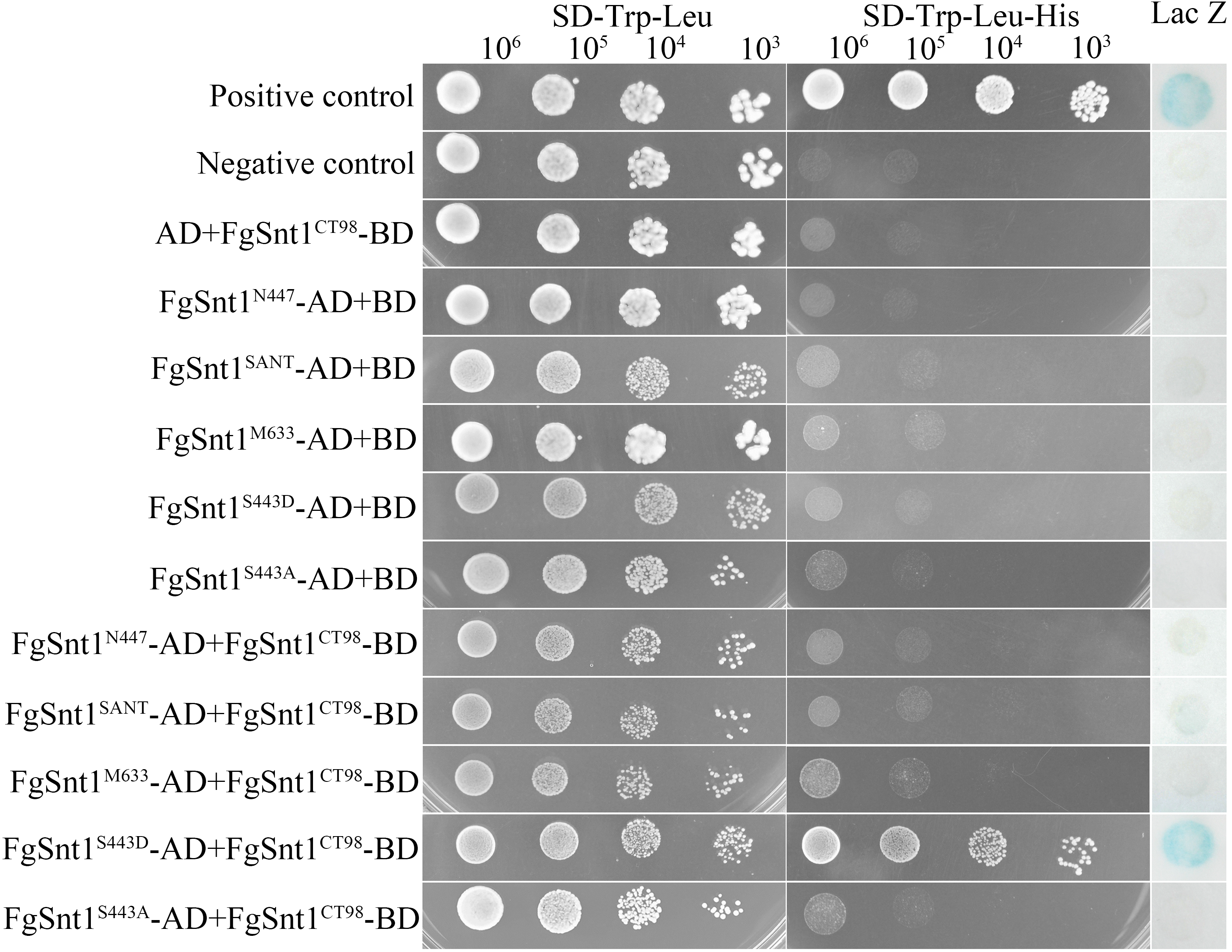

Supplement: S10 Fig — Yeast two-hybrid assays with cells expressing the empty prey or bait constructs as the negative controls for Fig 11. (TIF) [file pgen.1010510.s014.tif]
